# Nanoparticles in tumor microenvironment remodeling and cancer immunotherapy

**DOI:** 10.1186/s13045-024-01535-8

**Published:** 2024-04-02

**Authors:** Qiang Lu, Dongquan Kou, Shenghan Lou, Milad Ashrafizadeh, Amir Reza Aref, Israel Canadas, Yu Tian, Xiaojia Niu, Yuzhuo Wang, Pedram Torabian, Lingzhi Wang, Gautam Sethi, Vinay Tergaonkar, Franklin Tay, Zhennan Yuan, Peng Han

**Affiliations:** 1 Department of Thoracic Surgery, Tangdu Hospital, Air Force Medical University, 569 Xinsi Road, Xi’an, 710038 China; 2 Department of Rehabilitation Medicine, Chongqing Public Health Medical Center, Chongqing, China; 3 Department of Colorectal Surgery, Harbin Medical University Cancer Hospital, Harbin, China; 4 Department of General Surgery, Institute of Precision Diagnosis and Treatment of Digestive System Tumors, Carson International Cancer Center, Shenzhen University General Hospital, Shenzhen University, Shenzhen, 518055 Guangdong China; 5 Shanghai Institute of Cardiovascular Diseases, Zhongshan Hospital, Fudan University, Shanghai, 200032 China; 6 Department of Radiation Oncology, Shandong Cancer Hospital and Institute, Shandong First Medical University, Shandong Academy of Medical Sciences, Jinan, 250000 Shandong China; 7 Xsphera Biosciences, Translational Medicine Group, 6 Tide Street, Boston, MA 02210 USA; 8 Department of Medical Oncology, Dana-Farber Cancer Institute, Harvard Medical School, Boston, MA 02115 USA; 9 Blood Cell Development and Function Program, Fox Chase Cancer Center, Philadelphia, PA USA; 10 School of Public Health, Benedictine University, Lisle, USA; 11 Department of Urologic Sciences and Vancouver Prostate Centre, University of British Columbia, Vancouver, BC V6H3Z6 Canada; 12 Cumming School of Medicine, Arnie Charbonneau Cancer Research Institute, University of Calgary, Calgary, AB T2N 4Z6 Canada; 13 Department of Medical Sciences, University of Calgary, Calgary, AB T2N 4Z6 Canada; 14 Present Address: NUS Center for Cancer Research (N2CR), Yong Loo Lin School of Medicine, National University of Singapore, Singapore, 117599 Singapore; 15 Present Address: Department of Pharmacology, Yong Loo Lin School of Medicine, National University of Singapore, 16 Medical Drive, Singapore, 117600 Singapore; 16 Laboratory of NF-κB Signalling, Institute of Molecular and Cell Biology (IMCB), Agency for Science, Technology and Research (A*STAR), 61 Biopolis Drive, Proteos, 138673 Singapore, Republic of Singapore; 17 The Graduate School, Augusta University, 30912 Augusta, GA USA; 18 Department of Oncology Surgery, Harbin Medical University Cancer Hospital, Harbin, China; 19 Key Laboratory of Tumor Immunology in Heilongjiang, Harbin, China

**Keywords:** Bioengineered nanostructures; cancer immunotherapy, Immune evasion nanoparticles, Tumor microenvironment

## Abstract

Cancer immunotherapy and vaccine development have significantly improved the fight against cancers. Despite these advancements, challenges remain, particularly in the clinical delivery of immunomodulatory compounds. The tumor microenvironment (TME), comprising macrophages, fibroblasts, and immune cells, plays a crucial role in immune response modulation. Nanoparticles, engineered to reshape the TME, have shown promising results in enhancing immunotherapy by facilitating targeted delivery and immune modulation. These nanoparticles can suppress fibroblast activation, promote M1 macrophage polarization, aid dendritic cell maturation, and encourage T cell infiltration. Biomimetic nanoparticles further enhance immunotherapy by increasing the internalization of immunomodulatory agents in immune cells such as dendritic cells. Moreover, exosomes, whether naturally secreted by cells in the body or bioengineered, have been explored to regulate the TME and immune-related cells to affect cancer immunotherapy. Stimuli-responsive nanocarriers, activated by pH, redox, and light conditions, exhibit the potential to accelerate immunotherapy. The co-application of nanoparticles with immune checkpoint inhibitors is an emerging strategy to boost anti-tumor immunity. With their ability to induce long-term immunity, nanoarchitectures are promising structures in vaccine development. This review underscores the critical role of nanoparticles in overcoming current challenges and driving the advancement of cancer immunotherapy and TME modification.

## Introduction

Cancer cells are mainly suppressed by the complicated networks in the immune system, but tumors develop several mechanisms to evade anti-cancer immunity [1]. Hence, cancer immunotherapy has been introduced as a new mainstay to utilize the patient’s own immune system in cancer cell eradication. The cancer immunotherapy concept can be categorized into immune checkpoints-targeted therapy and the adoptive transfer of manipulated immune cells. Both of these strategies contribute to improving the immune system’s function in the identification and eradication of cancer cells [2]. A number of immune checkpoint inhibitors, including programmed cell death ligand 1 (PD-L1) or cytotoxic T-lymphocyte-associated protein 4 (CTLA-4) antibodies and agonists of costimulatory molecules have shown satisfactory results in clinics for the treatment of cancer patients, however, they still have a number of troublesome problems including low response rate, high cost and non-specific toxicity [3–5]. Another method is the adoptive transfer of cells, which uses genetically engineered cells such as chimeric antigen receptor (CAR)-T cells and others such as multipotent mesenchymal stem cells to affect the expression of a specific cytokine and other features of cells [6, 7]. Since the promising application of immune checkpoint blockade and CAR-T cell therapy, cancer immunotherapy has undergone significant advances. Cancer immunotherapy is now considered a powerful and innovative strategy in clinics compared to other conventional treatments such as surgery, radiotherapy, and chemotherapy. The most recent immunotherapeutic methods have utilized T cells for the induction of adaptive immune responses. On the other hand, studies have shown that a number of innate immune checkpoints with expression on the antigen-presenting cells (APCs) contribute to immune evasion. These checkpoints are capable of detecting and eradicating tumor cells through phagocytosis and suppressing innate immune response. The first line of the immune defense system is provided by innate immune cells such as macrophages, monocytes, and dendritic cells that act as APCs. They induce pro-inflammatory reactions to foreign attacks and contribute to the repair of damaged tissues. The cancer cells are able to express a number of signals known as “do not eat me” signals through expression of CD47 [8], CD24 [9], PD-L1 [10], the beta-2 microglobulin (β2M) subunit of MHC-I [11], stanniocalcin 1 (STC-1) [12] and GD2 [13] to evade the macrophage-mediated phagocytosis.

The application of cancer immunotherapy has been of importance in the treatment of both hematological and solid tumors [14]. In fact, immunotherapy has revolutionized cancer therapy, and it aims to apply immune checkpoint inhibitors, adoptive cell therapy, and vaccines to finally target the immune-oncology cycle for improving the activity of T lymphocytes in tumor suppression. All of these regimens should be applied in a cycle to accelerate the tumor antigen presentation by APCs [15]. APCs are the cells with the ability to capture, process, and present the exogenous antigens to T cells, and are primarily recognized through the expression of MHC-II and other co-stimulatory molecules. APCs are mainly comprised of dendritic cells, macrophages, and B cells. There are also other cells with expression of MHC-II such as thymic epithelial cells. Moreover, there are also other kinds of cells, such as eosinophils and basophils, with the ability of MHC-II expression upon stimulation [16, 17]. Regarding solid tumors, antigen uptake and presentation are mainly performed by macrophages and dendritic cells [18]. Although macrophages are the prevalent phagocytic cells in cancers, they lack the ability to migrate into lymph nodes and activate T cells [18]. Noteworthy, macrophages are considered a factor in impairing the T cell-mediated responses against tumors, and they reduce the response to immune checkpoint blockade, chemotherapy, and radiotherapy [19, 20]. On the other hand, the dendritic cells have a significant ability in lymph node migration to induce T cells for immunity [18, 21–24]. Moreover, increasing evidence has shown the ability of tumor-resident dendritic cells to stimulate T cell-mediated anti-cancer immune response [18, 24–28]. However, it should be noted that macrophages and other cells in the TME, including fibroblasts, can exert carcinogenic activity upon induction and their regulation is of importance for maximizing cancer immunotherapy [19, 29–36]. Stimulation of TME remodeling represents a beneficial strategy for cancer treatment and immunotherapy [37–43]. Although a significant number of studies advocate the potential of immunotherapy in cancer suppression [44–48], immunotherapy has faced its own problems, and the most prominent one is immune evasion. Regarding the mutations and dysregulation of molecular pathways in human tumors, the oncogenic pathways are activated, which can finally promote the survival of cancer cells and mediate their escape from cancer immunotherapy and immune surveillance.

In addition to immune resistance, current drugs used for cancer immunotherapy and TME remodeling lack targeting features. As a result, the nanoparticles have been introduced to facilitate anti-cancer immunity. Nanostructures can improve the retention time and provide the targeted delivery [49]. Furthermore, nanoparticles are capable of TME remodeling to disrupt an immunosuppressive environment. The distortion of blood vessels and high growth rate of tumor cells cause hypoxia in TME that mediates an immunosuppressive environment, characterized by an increase in the accumulation of immunosuppressive cells, including regulatory T cells (Tregs) and myeloid-derived suppressor cells (MDSCs), as well as secretion of a number of factors including vascular endothelial growth factor (VEGF) and transforming growth factor β (TGF-β). As a result, the function of dendritic cells is suppressed and macrophages are polarized into M2 phenotype. The nanoparticles can be specifically developed to target TME components and disrupt the immunosuppressive TME to improve the function in cancer immunotherapy. Moreover, nanoparticles can be considered as nano-scale delivery systems for drugs [50]. They can selectively accumulate in tumor tissue and enhance the retention time of drugs. Moreover, nanostructures demonstrate enhanced permeability and retention (EPR) effect, improving their accumulation in tumor sites because of leaky tumor vasculature and damaged lymphatic drainage [51, 52]. Furthermore, nanostructures can be functionalized with ligands, to specifically target the tumor and even the TME components [53–55]. As a result, nanoparticles have been introduced as new structures for potentiating cancer immunotherapy and overcoming immune evasion [56–63].

**Fig. 1:**
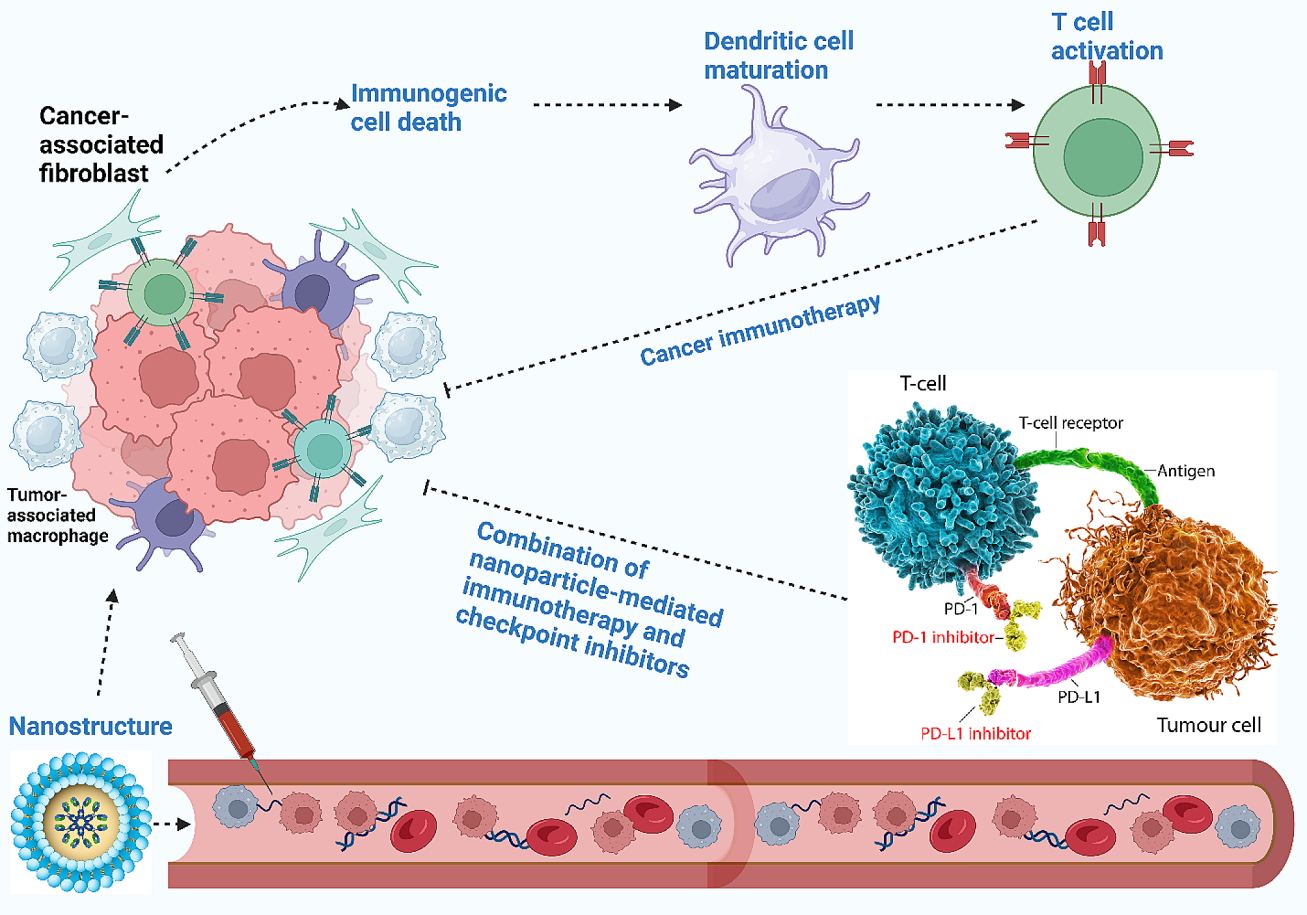
An overview of using nanoparticles in cancer immunotherapy. The nanoparticles circulate in blood, and upon reaching the tumor site, they re-educate several tumor microenvironment components, including cancer-associated fibroblasts and tumor-associated macrophages, to finally activate the immune system. Moreover, nanoparticles can stimulate immunogenic cell death to enhance the maturation of dendritic cells for the activation of immune cells, such as T cells, to enhance cancer immunotherapy. The co-application of nanoparticles with immune checkpoint inhibitors, such as PD-L1 blockers, can augment the potential of cancer immunotherapy

Amidst the current challenges in cancer immunotherapy, the need for improving cancer immunotherapy, and the promise of nanoparticles, the use of targeting systems has emerged as a novel strategy for immunotherapy enhancement and TME remodeling. The development of nanoparticles based on targeting TME and improving cancer immunotherapy can strengthen the potential for tumor eradication. Since the TME modulators suffer from targeted action, it is preferred to use targeted non-scale delivery systems for the regulation of TME and improving cancer immunotherapy. The present review focuses on the application of nanoparticles for TME remodeling and boosting cancer immunotherapy. The current review will first provide a comprehensive outline regarding TME components and then, immune evasion is discussed. Then, the potential of nanoparticles for TME re-education through targeting its components, including macrophages, is described. Moreover, biomimetic nanoparticles and their stimuli-responsive kinds for better tumor targeting are described. Since exosomes have emerged recently in cancer immunotherapy, the role of exosomes, both endogenous and bioengineered, in the regulation of the immune system for tumor suppression is discussed. Figure 1 provides an overview of using nanoparticles for cancer immunotherapy.

## Tumor microenvironment components

### Macrophages

Macrophages, renowned for their phagocytic nature, play a crucial role in the immune system. They participate in various physiological processes, including development and homeostasis. The phenotype and function of macrophages are intricately determined by their origin and polarization [64]. Initially believed to originate from hematopoietic stem cells and circulating monocytes [65], the recent studies reported that macrophages have an embryo-derived lineage, with precursors derived from erythro-myeloid progenitors in yolk sacs and fetal liver [65, 66]. Maintaining or enhancing the macrophage population is essential for these cells to function effectively [67, 68]. There are two strategies for the replenishment of macrophages: monocyte recruitment and increased proliferation in the form of tissue-resident macrophages for elevating self-renewal ability [67, 69].

In the TME, macrophages are referred to as tumor-associated macrophages (TAMs), constituting 50% of the tumor mass [70]. The TAMs engage in intricate interactions not only with cancer cells but also with natural killer (NK) cells, T cells, endothelial cells, and fibroblasts. The roles of TAMs extend to the regulation of cancer proliferation, invasion, and angiogenesis [71–73]. Macrophages have also been associated with the development of resistance to cancer therapies [74]. The TAMs primarily originate from the bone marrow or the yolk sac [75]. They can be polarized into two phenotypes. The M1 macrophages, induced by lipopolysaccharide and type 1 T helper cell (Th1)-derived cytokines, exhibit pro-inflammatory and anti-cancer functions [76]. The M2 macrophages, induced by Th2-derived cytokines like interleukin-4 (IL-4), IL-10, and IL-13, promote proliferation, invasion, and angiogenesis [76]. A delicate balance exists between M1 and M2 macrophages in vivo, influencing tumorigenesis and treatment outcomes [77, 78]. The anti-inflammatory nature of M2 macrophages accelerates cancer progression. The regulation of TAMs has been of importance for cancer immunotherapy. Currently, the strategies for targeting TMAs are based on controlling the origin, functional polarization, and phagocytic function of TAMs. Moreover, macrophages and monocytes have been engineered to mediate anti-cancer immunity. For this purpose, four distinct strategies have been exploited, including a decrease in TAM population, switching from M2 polarization into M1 phenotypes, controlling macrophage phagocytic signal, and bioengineering of macrophages for increasing phagocytosis [79]. Currently, the nanostructures have been widely applied to re-educate TAMs [80], change phagocytosis ability [81], suppress TAMs [82] and deliver drugs to TAMs [83] for cancer immunotherapy.

### Cancer-associated fibroblasts

Cancer-associated fibroblasts (CAFs) represent a diverse group of cells that infiltrate the TME. The CAFs are distinct from normal fibroblasts [84]. These cells play a pivotal role in tumorigenesis by inducing biochemical alterations and signaling network changes that accelerate tumor development [85]. Under specific conditions, CAFs may exhibit anti-cancer activities, contributing to tumor suppression [86].

The heterogeneity of CAFs arises from their diverse origins, including normal fibroblasts, epithelial cells, endothelial cells, peritumoral adipocytes, pericytes, hematopoietic stem cells, mesenchymal stem cells, and cancer stem cells [87]. Based on their functions, CAFs may be categorized into two groups: carcinogenic and anti-carcinogenic CAFs [88, 89]. Ohlund and colleagues identified two distinct subtypes of CAFs in pancreatic cancer: myofibroblasts (myCAFs) and inflammatory CAFs (iCAFs) [90]. The myCAFs, located near cancer cells, are stimulated by transforming growth factor-beta (TGF-β) and exhibit high levels of alpha-smooth muscle actin (α-SMA). In contrast, iCAFs are positioned further away from cancer cells. They demonstrate elevated α-SMA levels and the ability to secrete IL-6 and leukemia inhibitory factor [91].

Another subclass of CAFs, antigen-presenting CAFs (apCAFs), express biomarkers related to the MHC-II class and CD44, enabling them to stimulate CD^4+^ T cells in an antigen-dependent manner [92]. Additionally, there is a subtype known as restraining CAFs (rCAFs). Each of these subpopulations plays a distinct role in cancer. For example, iCAFs and myCAFs contribute to metabolic reprogramming and angiogenesis in cancer, respectively. The iCAFs can secrete growth factors, cytokines, and chemokines, including PD-L1/L2, Fas ligand, and others, that influence the regulation of the immune system. The myCAFs, on the other hand, contribute to extracellular matrix remodeling by enhancing collagen synthesis. The apCAFs are involved in stimulating CD^4+^ T cells for immune cell regulation, while rCAFs exhibit the ability to suppress tumorigenesis [93]. Regarding the importance of CAFs in tumorigenesis, targeting CAFs for cancer immunotherapy has been of importance. The nanostructures demonstrate high penetration and permeability in tumor sites, and can be utilized to regulate CAFs [94]. Moreover, nanoparticles can be utilized to engineer CAFs to act as APCs and stimulate antigen-specific CD^8+^ T cells in cancer immunotherapy [95]. Nanostructures can trigger clearance of activated and senescent CAFs [96], and regulation of CAFs by nanoparticles can disrupt cancer metastasis and invasion [97].

### Neutrophils

Up to 70% of circulating leukocytes are comprised of neutrophils [98], and are considered a first-line against pathogens [99]. Neutrophils have a short life and can persist in circulation for five days [100]. When there is tissue damage or infection, the epithelial cells secrete chemokines to recruit neutrophils. Upon this, neutrophils extravasate the blood circulation, enter damaged tissue to secrete a number of inflammatory cytokines, release neutrophil extracellular traps (NETs), and finally, phagocytose the pathogens or invading microorganisms [101]. NETs are vehicles for anti-microbial peptides and toxins [102, 103]. In cancer, there are two categories of tumor-associated neutrophils (TANs) similar to the Th1/Th2 pattern, including N1 and N2 with tumor-suppressor and tumor-promoting function, respectively. Tumor type and stage determine the phenotype of neutrophils in TME. During the first stages of tumorigenesis, neutrophils demonstrate an inflammatory phenotype, and as the cancer advances, the neutrophils achieve an immunosuppression phenotype [104]. Neutrophil-mediated inflammation regulation relies on the secretion of ROS and RNS. Moreover, the extracellular matrix can be re-configured by the neutrophils through the secretion of neutrophil elastase and matrix metalloproteinases. The neutrophils display the ability to stimulate angiogenesis through oncostatin-M, increase carcinogenesis through PGE2, and enhance metastasis of cancer through the release of ROS, RNS, NE and MMP-9. Noteworthy, the NETs have consisted of MMPs, cathepsin G, and NE [105, 106]. The function of these proteases is to mediate pro-inflammatory cytokine degradation and re-accumulate in TME for enhancement in tumorigenesis and metastasis [107]. In cancer patients, the plasticity of circulating neutrophils is of importance, known as high-density neutrophils (HDNs) or low-density neutrophils (LDNs), corresponding to N1 and N2 phenotypes, respectively. LDNs that have an immature phenotype, show prevalence in the circulation of many cancers and participate in carcinogenesis and metastasis [100]. In the field of cancer immunotherapy, the stimulation of N1 neutrophils can mediate toxic impacts on cancer cells [108]. Furthermore, the stimulation of Ly6Ehi neutrophils through the STING pathway can enhance sensitivity to anti-PD-1 therapy, and they can be utilized as predictors of cancer immunotherapy [109]. Therefore, the development of nanoparticles for targeting neutrophils in cancer immunotherapy is important.

### Natural killer cells and T cells

As innate lymphocytes, NK cells exhibit a shorter half-life compared to B and T cells, necessitating their replenishment from bone marrow progenitors [110]. The NK cells undergo linear differentiation, with highly proliferative immature NK cells differentiating into fully functional and granular effectors [111–113]. Enhancing the frequency, infiltration, and function of NK cells contributes to the improved survival of cancer patients [114–117]. This renders NK cells valuable in cancer immunotherapy. These group I innate lymphoid cells can rapidly target cells without prior sensitization [118], and express T-bet and Th1-related cytokines, including IFN-γ [119–121].

Upon maturation, NK cells migrate from the bone marrow to the blood and subsequently reside in peripheral tissues. Because of their capacity to move between lymphatic and non-lymphatic tissues, NK cells are distributed in numerous organs and tissues [122–124]. Mature NK cells acquire the capability to exert cytotoxic impacts on cancer cells or virus-infected cells [125]. Serving as contributors to the adaptive immune system, NK cells interact with other immune cells through the secretion of cytokines, growth factors, and chemokines [125]. These effects position NK cells as effective effectors in diseases such as cancer, infectious diseases, autoimmunity, and chronic inflammation [126–129].

Moreover, NK cells play a significant role in the innate immune system, providing surveillance in hematological cancers and cancer metastasis [110, 130, 131]. The increased infiltration of NK cells into the TME is positively associated with the prognosis of various cancer types, including melanoma, renal cell cancer, liver tumors, and breast cancer, among others [132–136].

The adaptive immune system is primarily shaped by T cells, providing effective defense against pathogens and cancers. Upon exposure to cytokines and co-stimulatory signals, naïve T cells undergo proliferation, differentiating into effector cells. Naïve CD^4+^ T cells can differentiate into T helper cells, including TH1, TH2, TH17, and TFH cells, to exert immune functions. The differentiation of naïve CD^8+^ T cells into effective CD^8+^ T cells enables these cells to combat infections and cancers through the release of IFN-γ, TNF-α, and cytotoxic molecules [137].

A challenge in cancer arises from T cell exhaustion. This phenomenon is mediated by various mechanisms, with the PD-1 axis being the most prominent. Upon antigen exposure, naïve T cells transform into effector T cells, with some undergoing cell death and others participating in tumor elimination. Antigen presentation can lead to the formation of stem cell memory T (TSCM) cells, which convert into TCM, TEM, and TRM. The TRM cells reside in the tissue, ready to respond to secondary stimulation, while TSCM and TCM possess self-renewal capacity, generating TEM and TE upon re-stimulation [138].

Signs of T cell exhaustion include the expression of inhibitory receptors, reduction in T cell function, and decreased proliferation. Exhausted T cells exhibit a unique epigenetic profile that may result in a differential or poor response to immunotherapy. Additionally, exhausted T cells experience metabolic dysregulation, including mitochondrial suppression and glycolysis inhibition [139]. The challenge in cancer therapy extends beyond T cell exhaustion, as their death and reduced proliferation can impair immune reactions. Targeting NK and T cells with nanoparticles has strengthened cancer immunotherapy. The nanoparticles with high uptake in NK cells, such as lipid-based nanoparticles, can be utilized to engineer NK cells [140]. Furthermore, nanostructures can be utilized for non-invasive tracking of NK cells, including their migration and biodistribution in tumor regions [141]. The expression levels of CCR4 and CXCR4 on the surface of NK cells can be changed by nanoparticles to improve their interaction with cancer cells [142]. Noteworthy, the nanoparticles can be designed to stimulate both NK and CD^8+^ T cells in cancer immunotherapy [143].

### Endothelial cells and pericytes

Endothelial cells form the inner lining of blood vessels. The biological functions of endothelial cells are crucial for preserving normal physiological conditions [144]. These cells play important roles in regulating blood clotting, vessel size, and immune functions to enhance blood fluidity, oxygen distribution, cell transport, and nutrient supply. Endothelial cells continuously secrete anticoagulant proteins to prevent clotting in vascular beds, maintaining homeostasis and ensuring blood flow and pressure at an appropriate level to deliver oxygen and nutrients to tissues [145–149].

Despite their essential physiological functions, endothelial cells have been implicated in cancer progression. Recent reviews have highlighted the role of endothelial cells in the tumor stroma [150, 151]. In the initial stages, endothelial cells induce angiogenesis to increase the presence of blood vessels in the primary tumor. These cells also function as a platform and site for membrane-bound factors and proteins, creating a TME conducive to cancer progression. These localized functions of endothelial cells also play a role in regulating angiocrine signaling at distant sites, influencing organ function. Furthermore, factors and proteins secreted by tumor cells can extend beyond tumor boundaries, affecting endothelial cells at distant sites and exerting systematic functions [152].

Understanding the functions and regulatory impacts of tumors beyond their sites is crucial, given that most cancer-related deaths result from invasion, thrombosis, and cachexia [153–155]. Proteins and factors secreted by tumor cells can induce changes in endothelial cells in the pre-metastatic niche, enhancing the dissemination of cancer cells and mediating angiogenesis. Additionally, these factors can lead to thrombosis in distant vasculature [156]. Endothelial pericytes have been recognized more than a century ago as microvasculature-associated mural cells [157]. These perivascularly positioned cells [158–160] are ubiquitously distributed in all vascularized tissues [161, 162]. Identification of pericytes requires immunostaining and the use of biomarkers and antigens to differentiate them from vascular smooth muscle cells, fibroblasts, and mesenchymal cells [157]. Initially considered inert cells contributing to physical vascular stability [163, 164], recent advances have illuminated their roles in both physiological and pathological conditions.

**Fig. 2:**
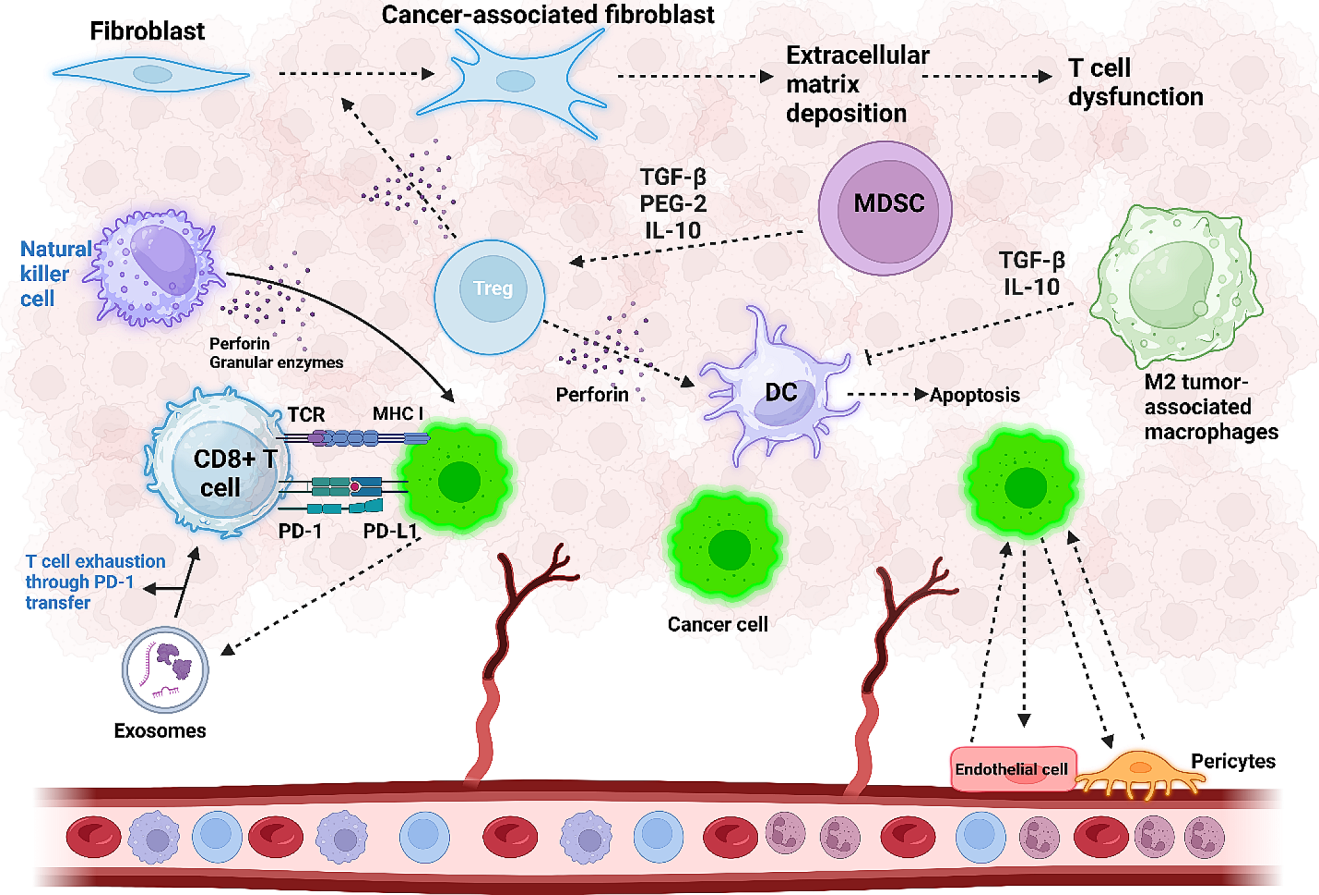
Cellular components that influence the tumor microenvironment (TME). Interactions within the TME play a crucial role in accelerating cancer progression. Cancer cells activate the PD-L1/PD-1 axis, leading to T cell exhaustion and impairment of T cell function. In addition, cancer cell-secreted exosomes that carry PD-1 contribute to T cell dysfunction, reducing proliferation and hindering proper function. Natural killer cells counteract tumorigenesis by secreting perforin and granular enzymes. Increased infiltration of Treg cells in the TME secretes TGF-β, inducing fibroblast transformation into cancer-associated fibroblasts (CAFs), promoting extracellular matrix deposition, and causing T cell dysfunction. Myeloid-derived suppressor cells (MDSCs) induce Treg cell formation in the TME through the secretion of PGE-2, IL-10, and TGF-β. Regulatory T cells (Treg), in turn, suppress the function of dendritic cells (DCs) by secreting perforin, leading to DC cell apoptosis. M2-polarized macrophages secrete TGF-β and IL-10, disrupting DC cell function. The interaction between endothelial cells and cancer cells results in angiogenesis, further enhancing cancer progression (created by Biorender.com)

Pericytes play a crucial role in regulating blood vessel development and modulating blood flow, coagulation, and vascular permeability [165]. The structure of capillaries includes endothelial cells, pericytes, the basement membrane, and vascular smooth muscle cells [166]. The primary function of pericyte function in cancer progression is stimulating angiogenesis in the TME [167]. The CD248 is capable of Wnt upregulation and increasing the levels of OPN and SERPINE1 in pericytes to cause angiogenesis and expedite cancer progression [168]. Additionally, pericyte contractility can be induced by the enzyme hexokinase 2 in glycolysis, leading to abnormalities in tumor blood vessels [169]. When present in the tumor site, RGS5-TGFβ-Smad2/3 creates an anti-apoptotic environment that accelerates cancer cell growth [170]. Figure 2 is a schematic representation of TME components.

### Myeloid-derived suppressor cells

The Myeloid-derived suppressor cells (MDSCs) are another type of cell present in TME. There are a number of arguments that MDSCs are a subtype of neutrophils [104] due to the presence of overlapping markers among MDSCs and TANs, making it challenging and problematic to distinguish them. There is still controversy regarding whether MDSCs represent a separate lineage of cells or are polarized immature neutrophils [171]. Overall, MDSCs are considered a heterogeneous population of cells with myeloid origin [172]. In spite of origination from myeloid progenitor cells, MDSCs and TANs are considered different cell types. Furthermore, MDSCs demonstrate several distinct features from neutrophils, including downregulation of CD16 and CD621 and upregulation of Arg1, CD66B, and CD11b [173, 174]. Furthermore, the studies have shown other subtypes of MDSCs including monocytic MDSCs (M-MDSCs), which are distinguished by a CD11b hi, LY6C hi, and LY6G lo phenotype, polymorphonuclear MDSCs (PMN-MDSCs), which display a CD11b hi, LY6C lo, and LY6G hi phenotype, and early stage MDSCs (eMDSCs) which are CD13- and CD14-, and CD33 + in humans [175, 176]. In TME, it is possible to observe both M-MDSCs and PMN-MDSCs, and compared to MDSCs, they demonstrate a suppressive phenotype [177]. The MDSCs suppress T cells and the innate immune system to create an immunosuppressive phenotype in TME [177]. MDSCs also contribute to the formation of pre-metastatic niches, can elevate stemness and angiogenesis, and promote metastasis through EMT induction and enhancing IL-6 secretion [178, 179]. There are also other factors in TME that can affect MDSCs. The HIF-1α, a marker of hypoxic TME, stimulates the differentiation of MDSCs into TAMs with carcinogenic function [180]. The metabolism of MDSCs in TME can be changed towards stimulation of fatty acid oxidation to enhance levels of Arg1 and NOS2 [181]. For cancer immunotherapy, the regulation of MDSCs can provide new insights, such as the downregulation of CCRK that disrupts the immunosuppression activity of MDSCs and promotes the potential of immune-checkpoint blockade therapy [182]. The nanostructures are able to reduce the population and function of MDSCs, impair MDSC-mediated immunosuppression and cause MDSC repolarization [183–185].

### Cytokines, chemokines and other factors

The immune cells present in the TME use the cytokines to send messages to other cells in an endocrine, paracrine or autocrine manner and provide intercellular communication [186]. Cytokines, also known as immunomodulatory agents, can be produced in physiological and pathological status, and various classes of cells, including adipocytes and tumor cells, can secrete them. The cytokines contribute to the cellular (type 1) and antibody-mediated (type 2) immunity as anti/pro-inflammatory and pro/anti-tumorigenic effectors that also rely on the TME. Cytokines can bind to the receptor on the surface of other cells to regulate their action and change the molecular pathways. There are different kinds of cytokines in TME, including chemokines, interleukins, adipokines, transforming growth factors (TGFs), tumor necrosis factor (TNF), colony-stimulating factors (CSFs), and interferons (IFN) that can act alone or in a synergistic way to affect immune system [187]. Chemokines are considered as chemoattractant cytokines for the recruitment of inflammatory cells, including leukocytes (monocytes, neutrophils), along with other kinds of cells, such as endothelial and epithelial cells [188]. Depending on the position of conserved cysteine residues, there are various classes of cytokines including CX3C, CXC, CC, or C chemokines [189]. Moreover, chemokines are able to interact with the G protein-linked transmembrane receptors known as chemokine receptors [190]. A number of chemokines, such as CXCL8 and CCL3, have an inflammatory function, and they recruit the cells via the inflammatory signs or/and homeostatic [191]. Interleukins (ILs) possess a low molecular weight and demonstrate pro- and anti-inflammatory functions. The immunocompetent cells, including T cells, granulocytes, monocytes, macrophages, adipocytes, and endothelial cells, can secrete ILs [192]. The ILs play a critical role in the development, differentiation, induction, maturation, migration, and adhesion of immune cells [193]. Adipokines (also known as adipocytokines) are cytokines that can be secreted by adipose tissue and consist of adipocytes, pre-adipocytes, macrophages, stromal cells, fibroblasts, and endothelial cells [194]. The adipokines are comprised of adipose tissue-specific cytokines (adiponectin, leptin) and other categories, including ILs, TNFs, and chemokines. Moreover, inflammation, energy metabolism, and fat distribution can be controlled by adipokines [195]. The adipokines also contribute to obesity-related inflammation to regulate metabolic diseases [196]. Adipocytes are critical regulators of tumorigenesis and metastasis [197]. According to the impact of adipokines on the immune system, there are two kinds, including pro-inflammatory, such as leptin, TNFα, interleukin-1β (IL-1β), interleukin-6 (IL-6), and interleukin-8 (IL-8), potentially linking adiposity and inflammation, and anti-inflammatory, such as interleukin-10 (IL-10) and adiponectin [197, 198]. A number of adipokines, such as adiponectin, demonstrate anti-carcinogenic function [198], while others, such as leptin, demonstrate carcinogenic function [199]. TGFs are a number of protein hormones that are overexpressed in human cancers and can modulate tumorigenesis and cancer growth. TGFα is a member of the EGF family with the potential to regulate epithelial development and cell proliferation and can modulate carcinogenesis and angiogenesis [200]. M2 macrophages and other kinds of cells, including cancer cells, can secrete TGF-β to modulate the function of T cells, NK cells, and macrophages present in TME, disrupting anti-cancer immunity and enhancing carcinogenesis [201]. IFN was discovered upon its function to interfere with viral growth [202]. The host cells secret IFNs, and they can regulate the immune system. The fibroblasts and monocytes are able to secrete type I IFNs such as IFN-α and IFN-β during the viral attack. Then, the expression of proteins with the ability to impair RNA and DNA replication is upregulated. The type II IFNs, including IFN-γ can be released by CD^8+^ T and Th1 cells to induce a number of cells, including NK cells, M1 macrophages, and CD^8+^ T cells for enhancing MHC I and II presentation, promoting the anti-cancer immunity [203].

### Enzymes

The changes in the expression level of enzymes are a feature of TME, and it can be exploited in a rational way to treat cancer [204]. Enzymes are a kind of protein or RNA that can facilitate chemical reactions [205]. The enzymes for catalyzing reactions are highly selective and under mild conditions, demonstrate the specific substrates to modulate biological and metabolic mechanisms [206]. The enzymes display a number of changes in expression in diseases such as TME [207]. The TME shows several enzyme secretions consisting of MMPs, hyaluronidase, γ-glutamyl transpeptidase, and esterase with higher expression in tumors compared to normal tissues [208, 209]. The proteases contribute to the degradation of proteins or peptide substrates. The oxidoreductases can mediate the catalysis of electron transfer from the reductant to the oxidant. Kinases provide phosphorylation to affect protein activity and phosphatases mediate dephosphorylation. A number of enzymes demonstrate upregulation such as MMP-2 [210]. In bladder tumors, the expression of HAse is enhanced compared to the normal tissues [211].

### Extracellular matrix components

The extracellular matrix (ECM) is comprised of collagen, fibronection, laminin, vitronectin, elastin, and other factors including growth factors, cytokines, and matrix metalloproteinases that contribute to the support of the epithelial cell structure [212, 213]. Various cells have the ability to secrete ECM components but they are mainly secreted by fibroblasts [214]. During cancer progression, ECM can be considered as an initiation factor. The composition of ECM can be different based on the type of cancer, such as gastric tumors, in which a lower degree of differentiation improves the abundance of ECM components, heightens cell metabolism, and increases metabolic reprogramming [215]. According to the proteomic analysis, there is no difference between ECM components in tumor and normal tissues, while their levels demonstrate changes that are manifested by enhancement in ECM proteins and reduction in basement membrane components modulating tumor angiogenesis, metastasis, and invasion [216]. The density of ECM components increases during tumor progression, and a number of factors, such as E-cadherin/β-catenin, demonstrate reduction, enhancing proliferation and metastasis of cancer cells [217]. The increase in matrix density can cause a kind of environmental stress to enhance carcinogenesis. The high-strength ECM can stimulate EMT to increase cancer progression and promote the infiltration of M2 polarized macrophages while it suppresses the function of CD^8+^ T cells [218, 219].

### Hypoxia

The presence of hypoxia is another feature of TME resulting from the high proliferation of tumor cells. The alterations in interstitial fluid pressure, decrease in pH, and enhancement in ROS generation can result from hypoxia [220]. In regions with hypoxia, there is high interstitial fluid pressure due to leaky vasculature and abnormal lymphatic drainage in the tumor [221]. Moreover, the hypoxia in TME can enhance the generation of lactic acid and carbonic acid through glycolysis induction, providing an acidic pH. The hypoxia-inducible factor (HIF) can induce carbonic anhydrase IX or XII to transform carbon dioxide and water into HCO3– that, upon diffusion out of the cell membrane, it enhances HCO3– levels in TME. Furthermore, the endosomal and lysosomal vesicles in tumor cells demonstrate more acidic pH compared to cytosolic pH [222]. The hypoxia TME displays a redox potential difference between intracellular space (reducing) and extracellular space (oxidizing). Such redox potential is vital for the development of smart and selective delivery of therapeutics [223]. The enzymatic reduction during hypoxia in TME can cause the metabolism of chemical factors, including nitro, quinones, aromatic N-oxides, aliphatic N-oxides, and transition metals [224]. Such a feature can be utilized to develop hypoxia-responsive structures for exploiting the hypoxic regions [225].

## Mechanisms of immune evasion in cancer and unanswered questions in cancer immunotherapy

The immune system has undergone a transformative evolution to combat cancer progression. However, immune responses can be suppressed, and tumor cells often employ mechanisms to evade these responses, a concept known as immune evasion. Recent studies have shed light on the major mechanisms contributing to the immune evasion of cancer cells.

Mutations within tumor cells can facilitate immune evasion. This is evidenced by the dysregulation of CD^8+^ T cells observed in clinical specimens harvested from ovarian cancer patients, accompanied by the activation of immunosuppressive signaling through TGF-β [226]. A well-known mechanism for inducing immune evasion is the upregulation of PD-L1. In hepatocellular carcinoma, USP22 expression increases via PRDM1, leading to diminished SPI1 degradation through USP22 upregulation. This, in turn, results in increased PD-L1 expression, promoting immune evasion [227].

Efforts to suppress PD-L1 have shown promise in disrupting immune evasion. RNF31, with its ability to downregulate PD-L1 by enhancing ubiquitination and degradation of YAP, improves the function of CD^8+^ T cells, offering potential in cancer immunotherapy [228]. While immune checkpoint inhibitors have significantly improved tumor suppression and immunotherapy potential, the efficacy of these treatments is compromised by mechanisms related to immune evasion.

In addition to the previously discussed mechanisms, Choi and colleagues [229] proposed that lactic acid, a byproduct of cancer cell metabolism, plays a crucial role in suppressing anti-cancer immunity. This hypothesis has been substantiated by further research, notably in the comprehensive review by Wang and colleagues [230]. Their findings indicate that the accumulation of lactic acid and the resulting acidic tumor microenvironment (TME) significantly impair anti-cancer immune responses. Notably, it has been demonstrated that the presence of lactic acid and the acidic conditions within the TME inhibit the function of various immune cells, including T cells and dendritic cells. This leads to an immunosuppressive environment that promotes tumor growth and metastasis. Such insights underscore the complex interplay between cancer cell metabolism and immune evasion, emphasizing the pivotal role of lactic acid and the acidic TME as key contributors to cancer progression.

Upregulation of inhibitors such as SUSD6, TMEM127, and WWP2 in MHC-I has been implicated in immune evasion. Downregulation of SUSD6 increases MHC-I antigen presentation, suppressing cancer progression in a CD^8+^ T cell-dependent manner. The mechanism involves SUSD6 forming a complex with TMEM127 and MHC-I to recruit WWP2 for lysosomal degradation of MHC-I, facilitating immune evasion [231].

MHC-I, a key factor in immune evasion, undergoes internalization and degradation by CEMIP, further reducing immune surveillance [232]. In addition, SOX4-induced T cell exhaustion mediated immune evasion. The interaction between cancer cells and CD^8+^ T cells, facilitated by Midkine, alters immune system responses [233]. In PTEN-deficient animal models, PI3Kβ downregulation results in STAT3 suppression, accelerating immune responses and revealing the potential of PI3Kβ in causing immune tolerance and evasion [234].

Chromosomal changes and deletions also play a role in immune evasion. Homozygous deletions affecting chromosome 9q21.3 impair the function of CDKN2A/B, hastening carcinogenesis. Half of these deletions affect the IFN gene cluster on chromosome 9q21.3, increasing the escape of tumor cells from CD^8+^ T cell surveillance [235].

Maintaining the balance of interferon responses is vital for cancer immunotherapy, as alterations in interferon and T cell levels can induce immune evasion. mTORC1 enhances B7-H3 expression, reducing T cell function and IFN-γ responses while increasing MHC-II expression [236]. One mechanism causing immune escape involves reducing the number of T cells, mediated by apoptosis induction. Extracellular galectin 4 stimulates T cell apoptosis, diminishing immune surveillance. Conversely, the downregulation of galectin 4 promotes M1 polarization of macrophages and enhances T cells and dendritic cells, disrupting immune escape [237].

The immune cells have shown potential in the identification and recognition of neoplastic cells possessing initiation mutations to suppress tumorigenesis [238]. Although the origination of a tumor is from a single transformed cell, the presence of genomic instability can cause the generation of cancer cells that are genetically heterogeneous with unique morphological and physiological characteristics. Moreover, the tumor cells have shown significant features in terms of surface molecule expression, proliferation and angiogenesis [239] resulting from the morphological and epigenetic plasticity. Hence, the cancer cells demonstrate expression of different antigens that may be tumor-specific or tumor-associated, differentiation antigens, and lectin-binding sites. Such antigens display uneven distribution on tumor subpopulations and can stimulate various immune responses [240]. Such tumor heterogeneity can significantly affect the genotype, gene expression, cellular morphology, metabolic activity, motility, and behaviors, including proliferation, antigen presentation, drug response, and metabolism [241]. Furthermore, this heterogeneity can be utilized for the diagnosis, therapeutic efficacy, and recognition of promising targets [242]. Such a heterogeneous nature of cancer cells can provide significant opportunities to escape from the function of immune cells. The tumor cells significantly proliferate in TME, which can cause hypoxia. The presence of hypoxia in TME recruits MDSCs and impairs the function of NK cells to provide a pre-metastatic niche [243], showing that cancer cells metastasize through suppressing immune surveillance. Upon surgical resection, the cancer cells undergo trauma, and such tumor cells can enhance the generation of cytokines and other factors, including IL-6, C-reactive protein (CRP), TNF-α, IL-1β to affect the immune system [244]. Therefore, the application of conventional therapies and the heterogeneous nature of cancer cells should be considered in immune evasion. The immune cells are able to suppress vulnerable cancer cells presenting tumor antigens [245], while the heterogeneous nature of cancer cells allows them to escape such action of the immune system. Furthermore, the tumor cells have shown capacity to induce apoptosis in tumor-specific cytotoxic T lymphocytes [246].

Therefore, since immune evasion commonly occurs in cancer, cancer immunotherapy has been introduced. Regarding cancer immunotherapy, there are a number of challenges that should be addressed. The first and most important challenge is regarding the fact that dominant drivers of cancer immunity should be highlighted. Moreover, more investigation should be directed towards understanding the function of organ-specific tumor immune context. Checkpoint inhibitors are commonly utilized for the treatment of human cancers, but there is still a long way towards understanding the molecular landscape of factors regulating primary versus secondary immune escape. There is a big question about whether it is better to use endogenous or synthetic immunity for the treatment of human cancers. Moreover, since cancer immunotherapy has been applied in clinics, there are also questions regarding the effective evaluation of cancer immunotherapy in clinical studies. One of the hopes is the advances in the field of biology highlighting the application of biomarkers and signatures for cancer immunotherapy. Therefore, precision medicine can significantly benefit by highlighting the signatures and developing strategies based on targeting accurate and efficient signatures for cancer immunotherapy. Another question is that various types of regimens for cancer immunotherapy have been developed, and comprehensive studies are required to be performed in improving long-term survival through a combination of such regimens. In order to optimize the process of cancer immunotherapy, responding to such concerns and questions can improve the potential for the treatment of cancer patients [247].

## Nanoparticles targeting tumor microenvironment components in cancer immunotherapy

### Nanoparticles targeting tumor-associated macrophages

To address the immunosuppressive role played by M2-polarized macrophages, the stimulation of M1 polarization through nanostructures emerges as a promising avenue for enhancing immunotherapy. A pivotal mechanism involves the development of genetically modified pristine cells, whose extracted cell membrane is utilized to coat and functionalize nanoparticles in cancer therapy. Biomimetic magnetic nanoparticles featuring gene-edited cell membranes demonstrate the capacity to target multiple pathways, thereby regulating macrophage polarization and suppressing tumorigenesis. Specifically, the presence of gene-edited cell membranes suppresses the CD44/SIRPα axis by upregulating SIRPα variants. Magnetic nanoparticles, forming the core, play a crucial role in re-educating and reprogramming macrophages, thereby accelerating cancer immunotherapy [248].

Changes in macrophages extend beyond polarization, and their role in regulating antigen processing is also significant. Certain clinically important pathways, such as STING, pose a challenge for targeting at the clinical level due to a lack of targeted delivery. By functioning as a STING agonist, ZnCDA encapsulates CDA and disrupts the endothelial barrier in cancer vasculature, facilitating penetration into the TME and tumor site. These nanoparticles target macrophages, enhancing antigen processing and expediting T-cell-related responses in cancer immunotherapy [249]. A number of nanoparticles have shown potential in changing the polarization of TAMs. In the context of M1 polarization of macrophages, different mechanisms are available for the induction of polarization of macrophages into the M1 phenotype. Ginseng-derived nanostructures with extracellular vesicle-like properties can stimulate the TLR4/MyD88 axis, resulting in increased M1 polarization of macrophages, elevated ROS levels, and induction of apoptosis in melanoma [250]. In fact, the M1 polarization of macrophages has been accompanied by apoptosis induction.

Although the primary focus of this section is to evaluate the role of nanoparticles in macrophage re-education, studies have demonstrated that membranes can be extracted from macrophages to coat and functionalize nanoparticles. This approach results in the development of biocompatible structures with stealth properties [251]. Such an approach can be used mutually in which nanoparticles are functionalized with macrophage membrane to improve their targeting ability towards macrophages and TME, and on the other hand, they can be designed for re-education of macrophages into M1 phenotype.

Targeting macrophages in cancer treatment is primarily driven by their immunosuppressive function. Despite the development of various immune response regulation strategies, such as phototherapy-induced immunotherapy, concerns persist regarding immunogenicity and inflammation induction. Therefore, it is crucial for nanoparticles to employ safe and biocompatible mechanisms to counteract macrophage-mediated immunosuppression. The biomimetic Fe_3_O_4_-SAS@PLT nanostructures, derived from sulfasalazine-loaded mesoporous magnetic nanostructures and functionalized with platelets, have been designed to suppress the glutamate-cystine antiporter system Xc-pathway in ferroptosis induction. This ferroptosis induction demonstrates synergistic effects with PD-L1 immune checkpoint immunotherapy, as observed in animal models. Notably, these biomimetic nanostructures induce ferroptosis, promoting M1 polarization of macrophages and disrupting the immunosuppressive TME [252].

When considering nanoparticles for modulating macrophages, especially for potential use in cancer immunotherapy at the clinical level, biocompatibility is as important as functionality. Lipid nanoparticles with cationic features have shown promise as carriers, delivering mRNA to targeted sites. Loading mRNA for re-educating macrophage polarization onto lipid nanoparticles creates safe and biocompatible nanostructures for cancer immunotherapy [253]. A significant advancement in utilizing nanoparticles for macrophage re-education involves functionalizing them with macrophage membranes to enhance efficacy. This hypothesis has been tested in experiments, demonstrating the potential of membranes derived from tumor-associated macrophages with immunomodulatory functions and antigen-homing affinity. These membranes were employed to functionalize upconversion nanostructures loaded with photosensitizers. Notably, tumor-associated macrophage membrane-functionalized nanoparticles suppress CSF1 and interactions between cancer cells and the tumor microenvironment, impairing tumorigenesis. Moreover, these nanoparticles stimulate photodynamic therapy by suppressing the M2 phenotype, enhancing M1 macrophage polarization, inducing immunogenic cell death, and improving the generation of T cells through enhanced antigen presentation [254].

Reorienting macrophages toward the M2 phenotype presents a hurdle in achieving successful immunotherapy. This polarization is chiefly instigated by tumor cell-secreted MCSF, resulting in the elevation of CSF1-R. Moreover, the heightened expression of SIRPα on myeloid cell surfaces activates SHP-1 and SHP-2 in macrophages, impeding immunotherapy by hampering phagocytosis. Moving beyond macrophage polarization, efforts are redirected to address macrophage activity failure. To augment macrophage phagocytosis, promising strategies involve the regulation of CSF1R and SHP2. Nanoparticles laden with CSF1R and SHP2 suppressors induce M1 macrophage polarization, boosting phagocytosis to impede tumorigenesis [255].

After elucidating the key mechanisms governing macrophage polarization and activity, the subsequent focus involves exploring nanoparticles with potential clinical applications. The FDA-approved ferumoxytol, an iron supplement and iron oxide nanostructure, serves dual roles as a drug delivery system and imaging agent. When co-cultured with macrophages for treating lung cancer metastasis, ferumoxytol upregulates caspase-3, inducing macrophages to express mRNAs for pro-inflammatory Th1-related responses. Ferumoxytol effectively suppresses tumor metastasis and proliferation while promoting M1 macrophage polarization to enhance the quality of cancer immunotherapy [256].

**Fig. 3:**
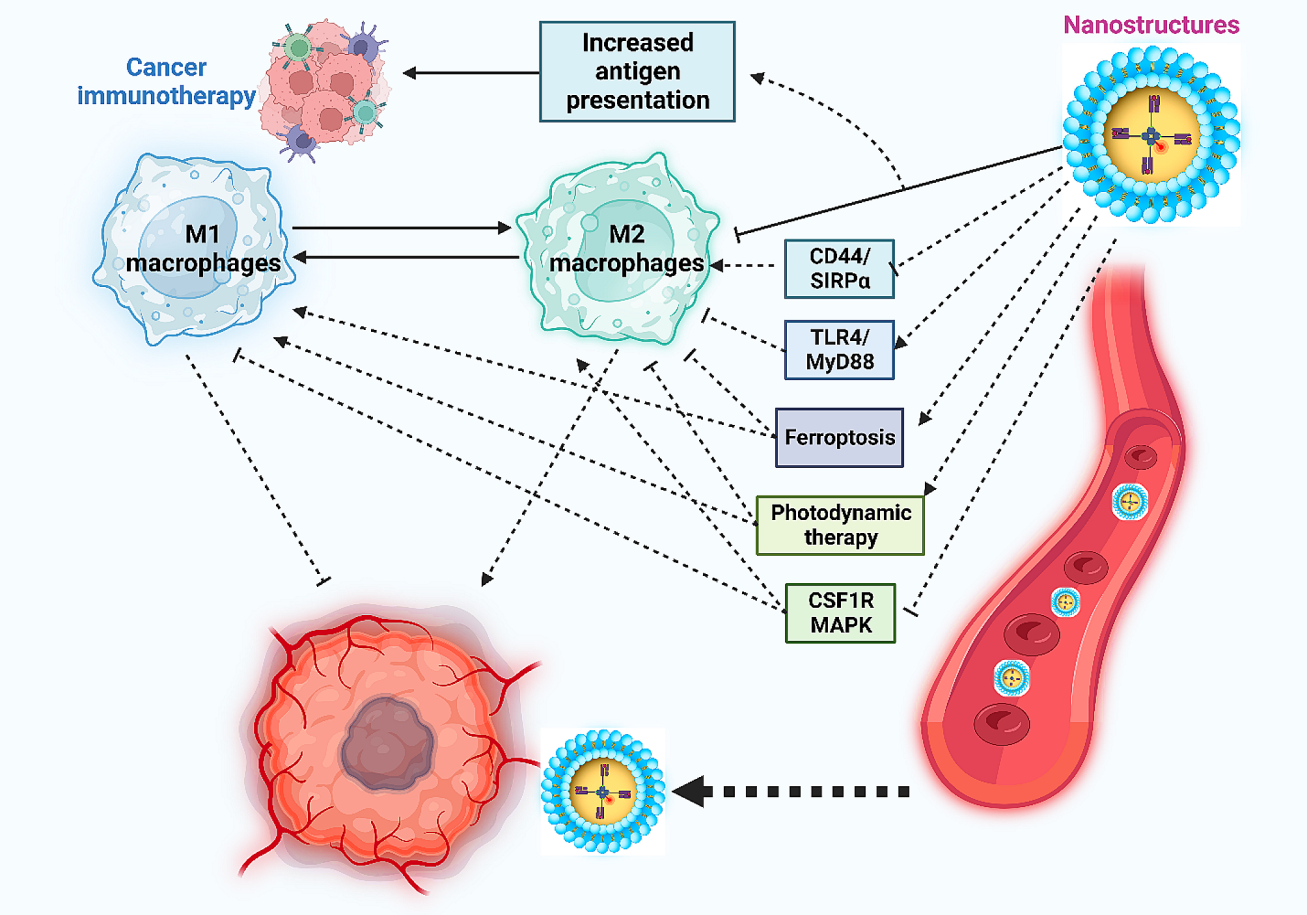
The impact of nanoparticles on macrophages, showcasing their potential to re-educate and impede cancer progression. These nanoparticles effectively target key mechanisms associated with the M2 polarization of tumor-associated macrophages. They inhibit CD44/SIRPα, CS1R, and MAPK, prompting the M1 polarization of macrophages. Additionally, nanocarriers activate the TLR4/MyD88 axis, contributing to increased M1 polarization of the tumor-associated macrophages. The nanoparticles further induce ferroptosis and photodynamic therapy, disrupting the polarization of these macrophages into the M2 phenotype (Created by Biorender.com)

A growing body of evidence supports the potential involvement of tumor-associated macrophages in the development of drug resistance [257, 258]. These macrophages play a role beyond immune system regulation, influencing the response to chemotherapy. Furin-based aggregated gold nanostructures capitalize on the “enhanced permeability and retention” effect, aggregating in breast cancer due to furin upregulation. This process suppresses exocytosis, leading to increased preferential accumulation at the tumor site. These nanoparticles also inhibit autophagy, promoting M1 macrophage education to counteract drug resistance [259]. Table 1 provides a concise overview of the applications of nanoparticles in macrophage re-education for cancer immunotherapy. Figure 3 provides an overview of the regulation of tumor-associated macrophages by nanoparticles in cancer immunotherapy.

**Table 1:** Nanoparticle-induced cancer immunotherapy through targeting macrophages

Nanoparticle	Cancer type/Cell line	Size (nm)/Zeta potential (mV)	Outcome	Reference
PEGylated liposomes	Breast cancer/4T1 cells Pancreatic cancer/ murine KPC1245 and KPC1242 cells	75 nm	Delivery of mannose and levamisole hydrochloride for glycolysis suppression and reducing mitochondrial energy metabolism Suppression of cancer proliferation Combination with radiotherapy impairs M2 polarization of macrophages and increases immune responses	[260]
Prodrug nanoparticles	Colorectal cancer/MC38 cells Breast cancer/MCF-7 cells	39 nm/-8.23 mV 263.2 nm/less than − 5 mV	Co-delivery of doxorubicin and R848 Modification of nanoparticles with bifunctional PD-1/PD-L1 peptide antagonist PCP Cleavage of nanoparticles with FAP-α in the tumor stroma Release of cargo in the tumor site stimulates immunogenic cell death and causes macrophage reprogramming	[261]
Lipid nanoparticles	Pancreatic cancer/KPC cells	122.4 nm/+27.82 mV	Loading lipid nanoparticles in injectable hydrogels Delivery of CCL5-siRNA by lipid nanoparticles to induce M1 polarization of macrophages and enhance T cell-induced immune responses	[262]
Upconversion nanostructures	Breast cancer/4T1 cells	39.5 ± 1.1 and 54.1 ± 1.3 nm/-19.7 mV and − 4.1 mV	Introduction of upconversion nanoparticles co-doped with perfluorocarbon (PFC)/chlorin e6 (Ce6) Targeted delivery of paclitaxel as a chemotherapy drug Increasing singlet oxygen production Stimulating M1 polarization of macrophage in accelerating pro-inflammatory cytokine release to impair breast cancer progression	[263]
Iron-chelated melanin-like nanocarriers	Colon and breast cancers/ CT26 and 4T1 cells	150 nm	Stimulating M1 polarization of macrophages and providing photothermal therapy, they accelerated tumor-associated antigen release to improve cancer immunotherapy	[264]
Supramolecular nanoparticles	Breast cancer/4T1 cells	190.1 nm/-17.1 mV	Suppression of CSF1R and MAPK to stimulate M1 polarization of macrophages	[265]
MIP-3β plasmid	Breast cancer/4T1 cells	90 nm/-2.1 mV	Increasing dendritic cell maturation and suppressing M2 polarization of macrophages	[266]
Au@PG nanocarriers	Lung cancer/ Lewis lung carcinoma cells	32.2 nm at 2.5 mM ONPG, 29.8 nm at 10 mM, 26.4 nm at 50 mM, and 18.3 nm at 75 mM	Polyaniline-based glycol-condensation on the nanostructures Switching M2 polarized macrophages into M1 polarized macrophages Nanoparticles with smaller sizes demonstrate higher efficacy in the macrophage re-education	[267]
CaCO3-loaded Au nanostructures	Macrophages/RAW 264.7 cells	32 nm	Elongating macrophage cell morphology Stimulation of M1 biomarker and inflammatory cytokines Inducing M2 polarization of macrophages	[268]
Polymeric nanocarriers	Osteosarcoma/K7M2 cells	98.4 nm/-14.3 mV	Biodegradable nanoparticles for delivery of regorafenib as vascular normalization compound Release of cargo upon laser irradiation of 808 nm and increasing hypoxia in TME Induction of the release of reactive oxygen species and mediation of immunogenic cell death Stimulation of M1 polarization of macrophages	[269]
Gadofullerene nanocarriers	Breast cancer/4T1 cells	68.1 nm/-37.7 mV	M1 polarization of macrophages and increasing infiltration of T lymphocytes in the TME for cancer suppression	[270]
DGL-ZA nanoparticles	Breast cancer/4T1 cells	123.1 nm/-13.4 mV	Potential cancer biodistribution, extravasation, and high tumor penetration Conjugation of dendrigraft poly-L-lysines as inducers of autophagy Macrophage regulation and increasing tumor-suppressor activity	[271]
Phosphatidylserine-modified nanoparticles	Melanoma/B16F10 cells	230 nm/at a range of 20–30 mV	Externalization of nanostructures occurs when they are exposed to the TME with upregulation of MMP2 Increasing depletion of tumular-associated macrophages in TME	[272]
Hyaluronic acid-functionalized nanoparticles	Non-small cell lung cancer	92 nm/-12 mV	Targeted delivery of miR-125b and increasing its transfection more than 6 times to induce M1 polarization and enhance iNOS levels	[273]
Trimethyl chitosan nanoparticles	Breast cancer/4T1 cells	120–160 nm/20 mV	Functionalization with mannose and glycocholic acid Delivery of SIRPα-siRNA and MUC1 pDNA Oral delivery of cargo pMUC1 increases macrophage phagocytosis ability and M1 polarization Increasing immunity by the SIRPα-siRNA	[274]

### Nanoparticles targeting cancer-associated fibroblasts

Nanoparticles play a crucial role in influencing cancer-associated fibroblasts (CAFs) within the cancer treatment landscape. Interactions between cancer cells and CAFs in the TME contribute to tumorigenesis, making it essential to explore nanoparticle applications in suppressing these interactions and impeding cancer progression. In ovarian cancer, ovarian cancer cells and TME cells promote the activation of ovarian CAFs. Gold nanoparticles with a size of 20 nm effectively disrupted this interaction, inhibiting CAF activation and offering potential in the treatment of ovarian cancer [275].

CAFs play a supportive role in tumor metastasis. Core-shell nanoparticles, with gold as the core and silver as the shell, were effective in suppressing osteopontin expression in CAFs, hindering cancer progression without impacting CAF biomarker expression [276]. Besides modulating CAF activation and secretions, nanostructures may also be used for targeted CAF destruction. Ultra-small iron oxide nanocarriers (6 nm in diameter) combined with low-frequency rotating magnetic fields induce mechanical forces, leading to CAF death and lysosomal disruption [277]. Targeting CAFs for destruction enhances nanoparticle internalization. Such a strategy addresses the challenge of a dense TME that hinders nanoparticle penetration. Ferritin nanocages loaded with the photosensitizer ZnF16Pc and modified with a single-chain variable fragment that targeted fibroblast activation protein, facilitated phototherapy to reduce CAFs and improve nanoparticle penetration into the tumor site [278].

Nanoparticles can serve dual functions in regulating CAFs and modulating immune responses. Poly(lactic-co-glycolic acid) (PLGA) nanoparticles functionalized with cancer cell membrane not only enhanced cancer cell-CAF interactions, but also increased antigen uptake, stimulating CD^8+^ and CD^4+^ T cells through MHC-I and MHC-II, thus promoting cancer immunotherapy [279]. The fibroblast activation protein, upregulated on CAF surfaces, represents a promising target in cancer immunotherapy. Nanoparticles functionalized with a single-chain variable fragment for ZnF16Pc delivery in cancer phototherapy lacked systemic toxicity. These functionalized nanoparticles suppressed cancer progression in both primary and distant sites by accelerating immune responses and promoting anti-CAF immunity [280].

Some nanoparticles are designed to respond to fibroblast activation protein as a CAF biomarker. Albumin nanostructures encapsulating paclitaxel and functionalized with CAP showed promise in targeting fibroblast activation protein in CAFs. Incorporation of the photosensitive compound IR-780 further enabled near-infrared laser irradiation for photothermal therapy, resulting in tumor suppression and improved deep tumor penetration [281]. The concept of specifically targeting CAFs using their biomarkers has significant potential in enhancing the fight against cancer.

### Nanoparticles targeting T cells

Nanoparticles, through targeted regulation of T cells, have emerged as a promising avenue for effective cancer immunotherapy [282–288]. Increasing the infiltration of CD^8+^ T cells and T helper cells in the TME is crucial for TME remodeling and activating the immune system against cancer progression. Nanoparticles such as manganese zinc sulfide nanostructures play a pivotal role in mediating this effect [289]. A noteworthy trend in recent years involves the integration of immunotherapy with other therapeutic modalities like chemotherapy or phototherapy. Hybrid prodrug nanocarriers carrying cisplatin and camptothecin, stimulate the cGAS/STING axis and induce DNA damage. Additionally, these prodrug nanocarriers enhance CD^8+^ T cell infiltration in the TME, improving immunotherapy outcomes for colorectal cancer. These hybrid nanocarriers possess a responsive feature to reactive oxygen species (ROS) and are constructed from mPEG2k-DSPE and other polymers [290]. The mPEG/PLGA/PLL nanocarriers, delivering CD155-siRNA and modified with PD-L1 antibodies, can simultaneously suppress CD155 and PD-L1, avoiding immune evasion. They enhance CD^8+^ T cell infiltration and induce immunogenic cell death in breast cancer therapy [291].

Developing an effective anti-cancer vaccine requires nanoparticles that can induce systemic immunity. MnO2-melittin nanostructures, responsive to changes in the TME, serve as promising vaccines by triggering systemic immune responses. These nanostructures induce cancer cell death through the Fenton reaction in the TME, activate the cGAS/STING axis, and enhance antigen-presenting cell maturation. Furthermore, MnO2-melittin nanoparticles stimulate systemic immune reactions, including the promotion of T cells and increased levels of pro-inflammatory cytokines and chemokines [292].

Combining chemotherapy with phototherapy is another strategy to expedite tumor suppression. Prodrug nanocarriers, developed from hyaluronic acid and adamantine-conjugated heterodimers of PPa and JQ1, target CD44-overexpressed pancreatic cancer cells. This combination of phototherapy and immunotherapy increases T lymphocyte infiltration. Moreover, JQ1 suppresses phototherapy-induced immune evasion by downregulating c-Myc and PD-L1, resulting in significant tumor suppression [293].

As cancer development is a gradual process, effective treatment should focus on providing long-term immunity. The use of cancer vaccines has significantly increased in recent years; however, a major challenge remains in the targeted delivery of cargo, including antigens and adjuvants. To address this issue, glycosylated poly(lactic-co-glycolic acid) (PLGA) nanocarriers have been developed for the delivery of the ovalbumin antigen and CpG as an adjuvant in cancer vaccination. The surface of the nanostructures is modified with galactose or mannose. These nanoparticles possess high loading ability and sustained release, which are key features for the development of cancer vaccines. They stimulate dendritic cell maturation, promote antigen uptake, and enhance CD^4+^ T cell levels, leading to increased infiltration of CD^8+^ T cells in cancer immunotherapy [294].

An innovative approach in cancer therapy involves developing nanoparticles that mimic pathogens to induce a robust immune response. *Saccharomyces cerevisiae* (yeast)-based nanocarriers function as nano-pathogen-associated molecular patterns (nano-PAMPs) and, through the induction of Dectin-2 and TLR-4, enhance TH17 responses, contributing to anti-cancer immunity [295]. Stimulation of T helper cells has proven effective in cancer immunotherapy. Chondroitin sulfate-modified nanostructures conjugated with glycolic acid or mannose, along with cationic liposomes loaded with ovalbumin, can stimulate the maturation of dendritic cells and evoke T helper type I and II responses [296]. In many cases, nanoparticles not only stimulate T cell infiltration, but also accelerate the maturation of dendritic cells, contributing to cancer immunotherapy [297]. Recognizing the role of epigenetic changes in immune dysfunction, the delivery of miRNAs has been explored in cancer immunotherapy. Lipid nanoparticles delivering anti-miR-21 have demonstrated the ability to stimulate M1 polarization of macrophages and enhance the infiltration of CD^8+^ T cells [298].

Nanoparticles have been employed for targeted regulation of immunosuppressive Treg cells in cancer treatment, aiming to enhance immunotherapy potential. For example, PLGA nanoparticles with antigen-capturing capabilities have been developed for this purpose. These nanoparticles primarily elevate the CD^8+^ T cell count, consequently increasing the ratio of cytotoxic T cells to Treg cells [299]. By augmenting this ratio, the negative impact of Treg cells on immune responses can be alleviated. For enhanced cargo delivery, layer-by-layer nanostructures, composed of GITR/PLGA and modified with PLG and PLH that are responsive to the TME pH, have been designed to deliver IR780 dye. Subsequent irradiation with a 808 nm laser promotes the maturation of dendritic cells, thereby increasing the activity of CD^8+^ and CD^4+^ T cells in cancer immunotherapy. Notably, these nanoparticles exhibit a suppressive effect on Treg cell function, contributing positively to immune reactions [300].

Several widely used chemotherapeutic drugs, including doxorubicin, face limitations such as low tumor site accumulation and the development of drug resistance. Prodrug nanocarriers based on doxorubicin and indoximod have been developed to suppress the IDO pathway. These prodrug nanocarriers induce immunogenic cell death, enhance the infiltration of cytotoxic T cells (CD^8+^ T cells), and suppress Treg cells, MDSCs, and TAMs in the TME, thereby effectively promoting T cell/Treg cell ratio for cancer immunotherapy [301].

Co-delivery strategies have been used to improve cancer immunotherapy. Metformin, a compound utilized for cancer immunotherapy, has shown promise in re-educating the TME and enhancing macrophage phagocytosis activity. Co-assembled prodrug nanoparticles, designed with hyaluronic acid-cisplatin/polystyrene-polymetformin, effectively co-deliver metformin and cisplatin. With a size of 166.5 nm and a zeta potential of -17.4 mV, these nanoparticles exhibit high potential in cancer immunotherapy. They induce apoptosis through PARP upregulation, enhance cisplatin sensitivity by suppressing ERCC1, and modulate AMPKα/mTOR pathways to increase CD^8+^ and CD^4+^ T cells, and reduce Treg cell numbers [302].

**Fig. 4:**
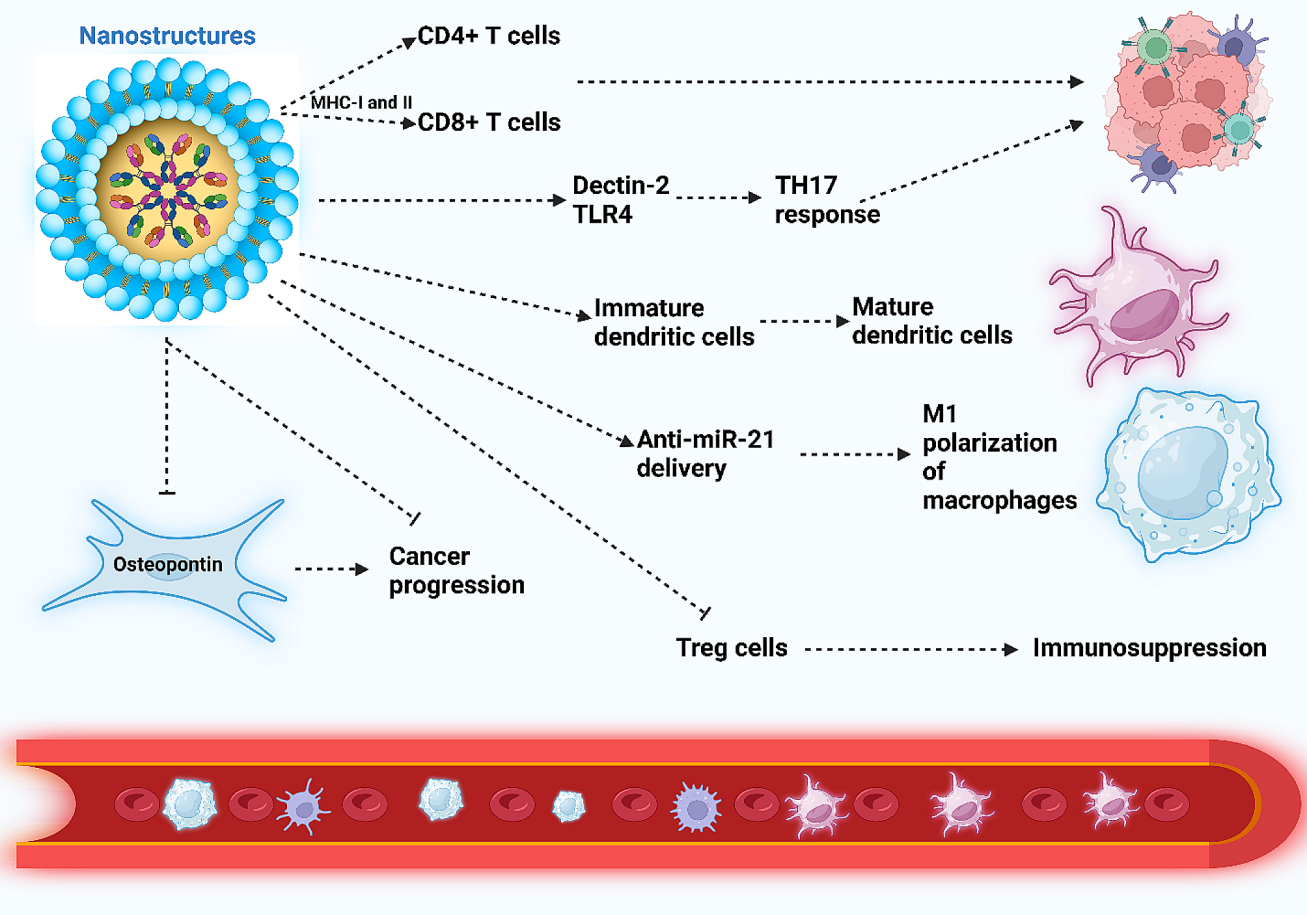
Nanoparticles orchestrating immune cells and cancer-associated fibroblasts (CAFs). Nanoparticles elevate antigen presentation via MHC-I and MHC-II, stimulating CD^4+^ and CD^8+^ T cells, thereby facilitating cancer immunotherapy. The nanostructures amplify Dectin-2 and TLR-4 levels, fostering TH17 responses for effective cancer immunotherapy. Additionally, they boost dendritic cell maturation and, through the delivery of anti-miR-21, induce polarization of macrophages into the M1 phenotype. The nanoparticles’ downregulation of osteopontin in CAFs disrupts cancer progression. Moreover, these nanoparticles suppress Treg cells, preventing immunosuppression (Created by Biorender.com)

Unmodified nanoparticles exhibit poor specific targeting of Treg cells. This prompted the use of nanocarrier functionalization. Hybrid nanocarriers functionalized with tLyp1 peptide have been developed to suppress STAT3 and STAT5, reducing Treg cell numbers and increasing the infiltration of CD^8+^ T cells in the TME [303]. The functionalized nanoparticles contribute to tumor suppression by increasing the infiltration of dendritic cells, CD^8+^ T, and natural killer cells, while reducing Treg and MDSC cells [304]. Furthermore, polymerosomes have been shown to stimulate the STING axis and enhance the infiltration and proliferation of T cells in cancer immunotherapy [57]. Table 2 summarizes the application of nanoparticles for the regulation of T cells in cancer therapy. Figure 4 demonstrates the role of nanoparticles in the regulation of CAFs, T cells, and Treg cells.

**Table 2:** Nanoparticle-mediated T cell regulation in cancer therapy

Nanoparticle	Cancer type/Cell line	Size (nm)/Zeta potential (mV)	Highlights	Reference
Polymeric nanoparticles	Lung cancer/LLC cells	75.9 ± 0.98 nm/32.5 ± 1.5 mv	ROS-responsive nanocarriers for the co-delivery of FGL1- and PD-L1-siRNA Development of nanoparticles from poly-l-lysine-thioketal and modified cis-aconitate to facilitate endosomal escape Functionalization of nanoparticles with iRGD peptide Enhancing infiltration of CD^4+^ and CD^8+^ T cells in cancer immunotherapy	[305]
Chiral nanoparticles	Lymphoma/EG7.OVA cells	-	Stimulation of NK and CD^8+^ T cells	[306]
Biomimetic nanoparticles	Colon cancer/CT26 cells	-	The phospholipid nanoparticles (PL1) can provide targeted delivery of mRNA (CD137 or OX40) in the stimulation of T cells	[307]
Cisplatin nanoparticles	Lung cancer/LLC	14.4 ± 3.3 nm/-12.8 mV	Enhancing CD^8+^ T cell priming through elevating antigen presentation and providing T cell crosstalk	[308]
Lipid nanoparticles	Colon cancer/MC38 cells	-	Stimulation of CD^8+^ T cells and reprogramming TME to disrupt the proliferation of cancer cells	[309]
Endogenous antigen-carrying nanoparticles	Breast cancer/4T1 cells	−15 ± 3.3 mV	Increasing proliferation of CD^4+^ and CD^8+^ T cells and promoting the ratio of cytotoxic T cells compared to Treg cells	[310]
Cationic polymeric nanostructures	Melanoma/B16F10 cells	163.9 ± 0.61 nm, 523.9 ± 15 nm and 1278.3 ± 27 nm/less than 60 mV	Development of nanocarriers based on polyadmidoamine dendrimers and poly(d,l-lactic-co-glycolic acid) Development of cancer vaccine Enhancing the number of T cells in the peripheral blood	[311]
Platelet	Breast cancer/4T1 cells	−38.0 ± 0.4 mV	Co-delivery of anti-PD-L1 antibodies and iron oxide nanoparticles as photothermal agents in cancer therapy Stimulation of necrosis through phototherapy Stimulation of innate immune responses Promoting infiltration of CD^4+^ and CD^8+^ T cells	[312]
Bacterial membrane-coated nanoparticles	Melanoma/B78 cells	207 nm/-11 mV	Comprised of PC7A/CpG core with immune system induction ability The presence of bacterial membrane and imide groups can increase antigen retrieval Capturing neoantigens and their presentation to dendritic cells Stimulation of T cell responses	[313]
Photo-responsive prodrug nanoparticles	Colon cancer/CT26 cells	88.1–119.2 nm	Delivery of VPF as photosensitizer, FRRG and doxorubicin Stimulation of immunogenic cell death ERP effect Maturation of dendritic cells for cross-presenting of antigens to T cells	[314]
K3ZrF7:Yb/Er upconversion nanocarriers	Breast cancer/4T1 cells	20 nm	Increasing ROS levels Capase-1 upregulation Gasdermin D cleavage IL-1β maturity Cytolysis induction Increasing dendritic cell maturation and promoting number of effector-memory T cells	[315]
Prodrug nanoparticles	Colon cancer/CT26 cells	70 nm/-17 mV	Targeted delivery of camptothecin and assembling with PEGylated lipids Increasing half-life and blood circulation Enhancing infiltration of CD^8+^ T cells	[316]
Lipid-coated calcium phosphate nanoparticles	Melanoma/B16F10 cells	30 nm/-20 mV	Apoptosis induction Acceleration of immunosuppression Polarization of macrophages into M1 phenotype Increasing CD^8+^ T cells	[317]

### Nanoparticles regulating hypoxia

In each tumor, the levels of oxygen are different [318]. The oxygen insufficiency in tumor tissue generally ranges from more or less anoxic state (almost no oxygen) to 60 mm Hg (8% oxygen). In spite of this, the tumor cells demonstrate a specific condition known as hypoxia in which oxygen levels fluctuate from anoxia to 7.5 mm Hg (about 1% oxygen) [319]. Hypoxia can be considered a reliable biomarker, since it promotes the progression of tumor cells and can cause therapy resistance [320]. Along with tumorigenesis, the hypoxia in cancer enhances, and it shows some coordination with angiogenesis, proliferation, and metastasis. Hypoxia is able to enhance the levels of CCL22, CCL28 and increases the accumulation of MDSCs and Tregs to mediate immunosuppressive TME [321–323]. Furthermore, hypoxia has been shown to be a factor involved in immune resistance [324]. Metformin is able to improve cancer immunotherapy by impairing the function of hypoxia in impairing CD^8+^ T cells [325]. Exercise has been shown as a mechanism for apoptosis induction and decreasing the proliferation of cancer cells. Moreover, exercise can ameliorate hypoxia, and enhance the function of T cells and reduces levels of Treg cells in cancer immunotherapy [326]. Hypoxia has been also shown as a mechanism in increasing M2 polarization of macrophages and secretion of factors with immunosuppressive function, including VEGF and TGF-β. Moreover, hypoxia has been suggested to cause therapy resistance, especially during photodynamic therapy and radiation in which oxygen molecules are required for cancer suppression [180, 327, 328].

Therefore, the function of hypoxia in cancer immunotherapy is of importance [329]. Hypoxia can be exploited by the nanoparticles for improving their specificity and recently, the hypoxia-responsive nanostructures have been designed for cancer immunotherapy [330–332]. However, most of the attention has been paid to the regulation of hypoxia in cancer immunotherapy. The biodegradable NIR-II pseudo conjugate polymeric nanostructures can regulate hypoxia in cancer immunotherapy. These nanostructures can deliver regorafenib and respond to 808 nm laser irradiation to release drugs for the reduction of cancer hypoxia through vascular normalization, allowing for oxygen entrance into tumors to increase ROS generation, mediating immunogenic cell death (ICD) for cancer immunotherapy. Moreover, these nanoparticles reprogram macrophages from M2 to M1 [269]. In another effort, albumin-based nanostructures have been developed for the co-delivery of IR780, NLG919, and hypoxia-activated prodrug tirapazamine (TPZ) in synergistic tumor suppression. Exposure to nanoparticles to NIR irradiation mediates the generation of ^1^O_2_ to trigger the release of ROS-responsive linker for TPZ release, causing chemotherapy through enhancing tumor hypoxia. Moreover, these nanostructures stimulate ICD to enhance the activity of cytotoxicity of T lymphocytes [333]. Doping the nanoparticles with Mn^2+^ can alleviate hypoxia and increase cGAS sensitivity, inducing the cGAS/STING pathway, causing macrophage re-education, and increasing the maturation of dendritic cells [334]. In a number of cases, the hypoxia is boosted in the TME to promote the release of drugs from nanoparticles for cancer immunotherapy [335]. Furthermore, macrophage-mimetic microalgae and liposomes have been conjugated to suppress autophagy and reduce hypoxia in cancer immunotherapy [336]. Regarding autophagy regulation, it should be highlighted that autophagy has a dual function in cancer and can exert carcinogenic and anti-carcinogenic functions, complicating its regulation in cancer therapy [337, 338]. According to these studies, the regulation of hypoxia by nanoparticles can pave the new gate for cancer immunotherapy [339–342].

### Nanoparticles targeting myeloid-derived suppressor cells

The infiltration of MDSCs is against anti-cancer immunity since it impairs T-cell proliferation and enhances the differentiation of Treg cells [343]. MDSCs are consideredimmature myeloid cells with a heterogeneous nature providing, an immunosuppressive TME [172]. Overall, MDSCs utilize three distinct mechanisms to cause immunosuppression. In the first method, the arginase 1 and iNOS undergo upregulation in MDSCs, and they can deplete _L_-arginine which is vital for the proliferation of T cells and CD3 ζ-chain formation of TCR. However, enhancement in arginase 1 activity and iNOS can suppress the proliferation and function of T cells [344–347]. In the second method, the MDSCs can enhance the generation of ROS and RNS to mediate dysfunction in T cells [348–350]. The ROS and peroxynitrite derived from MDSCs can cause post-transcriptional alterations in TCR and CD8 to interfere with the function of peripheral CD^8+^ T cells and cause antigen-specific tolerance in these cells through impairing binding affinity to phosphorylated MHC molecules [348]. In the third and final way, MDSCs are able to enhance Treg cell differentiation to disrupt anti-cancer immunity [172, 351]. Upon that, Treg cells secrete a number of inhibitory cytokines such as IL-10, IL-35, and TGF-β to interfere with the proper function of the immune system [352, 353]. Therefore, targeting MDSCs is critically vital for anti-cancer immunity. The intravenous administration of DNA thioaptamer-functionalized liposomes can cause specific targeting of TME, particularly MDSCs. Moreover, such liposomes provided targeted delivery of doxorubicin in breast cancer animal models to enhance infiltration of CD^8+^ T cells and diminish MDSCs [354]. Notably, there are different kinds of immune response-related molecules in TME, including IL-1β [355, 356], IL-6 [357], prostaglandin E2 [358], VEGF [359], and IFN-γ [351] that disrupt the differentiation procedure to increase the accumulation of immature myeloid cells [360]. As a result, one of the promising strategies can be targeting MDSCs for mediating their differentiation into other kinds of immune cells. In line with this, the lipid-coated biodegradable hollow mesoporous silica nanostructures have been introduced to regulate MDSC differentiation. Such nanostructures are able to co-deliver IL-2 and all-trans retinoic acid to trigger the MDSC differentiation into mature dendritic cells, macrophages and granulocytes. These nanoparticles showed significant capacity in enhancing the number of mature dendritic cells and decreasing MDSCs. Furthermore, these nanoparticles stimulated CD^4+^ and CD^8+^ T cells and increased the secretion of IL-12 and TNF-α as anti-tumor cytokines [361].

### Nanoparticles for delivery of cargo into antigen-presenting cells and lymph nodes

One of the prominent problems in cancer eradication using immunotherapy is the lack of effective and proper delivery into APCs and lymph nodes. The nanoparticles have opened a new gate for the delivery of immunotherapeutics into APCs and lymph nodes [362]. Noteworthy, a number of nanostructures based on their design, demonstrate the immunostimulatory impact, and even in lack of delivery of cargo, they can stimulate T and B cell responses [363, 364]. The tumor antigens have been conjugated into nanostructures, and upon injection into OVA-expressing melanoma, thymoma, or lymphoma-bearing mice, they caused significant anti-cancer immunity [365, 366]. Furthermore, such model antigens conjugated into nanostructures triggered T cell and antibody responses against lymphoma or colon tumors to impair cancer growth and enhance the survival of animal models [366–368]. The particle size has been considered an important factor in this case, as small virus-size particles (≤ 40 nm) can reach the lymph nodes and demonstrate high cellular uptake by dendritic cells. Then, the peptide presentation from coated antigen by dendritic cell-related MHC class I molecules occurs and the stimulation of CD^8+^ T cells is observed [369, 370]. The endocytosis of such nanostructures by dendritic cells can stimulate the danger-sensing pathway in dendritic cells and mature them for immunogenic APCs [371]. The tumor antigen should reach the tumor-draining lymph nodes to be absorbed by professional APC cells such as dendritic cells, and then, their presentation to T cells occurs. The tumor-specific T cells have been found in lymph nodes. However, the dendritic cells in tumor-draining lymph nodes demonstrate an immature/inactive form that compromises their ability to induce anti-tumor T cell responses [372–379]. The nanoparticle-bound cytosine-phosphate-guanine (CpG) oligonucleotides (an adjuvant) have been shown to accumulate in tumor-draining lymph nodes in melanoma to induce dendritic cells [380].

### Nanoparticles targeting tumor cells

A high number of nanostructure-based methods need tumor infiltration by nanoparticles [381]. The clinical implication of EPR [382, 383] is still under question and a controversial debate, and there is a discussion in which only a small proportion of administered nanostructures can reach the tumor tissues, lacking clinical importance and therapeutic value in the clinical setting [384]. Therefore, significant efforts have been made to improve the ability of nanoparticles to reach tumor tissues and optimize the nanostructures in a way to control biological interactions due to the complicated nature of TME resulting from irregular vascular distribution, high tumor interstitial fluid pressure, low blood flow, dense EZN and high number of stroma cells. Therefore, the strategies should be directed towards enhancing the entry of nanostructures into tumor tissue that can be obtained through improving tumor perfusion, elevating tumor vasculature permeability, and mediating ECM remodeling. As an example, the nanostructures applied for ECM degradation or restoring tumor vasculature into normal condition [385] can mediate TME priming to provide desirable immune reactions, reversing immunosuppressive TME and enhancing anti-cancer immunity [386]. In cases where tumors are accessible, the intratumoral administration of nanoparticles is preferred into systemic injection to enhance accumulation at the tumor region. The proper accumulation of nanoparticles in TME and the release of therapeutic cargo can enhance tumor suppression, while it reduces the adverse impacts. A number of clinical studies have suggested the intratumoral injection of immunotherapeutic compounds [387, 388] which has also been confirmed in pre-clinical studies upon intratumoral injection of immune checkpoint inhibitors [389]. The nanoparticles optimized to bind into ECM or cancer cells can enhance the accumulation of these structures in the tumor region [390] and provide a new insight into the effective delivery of therapeutics into tumor cells or TME.

## Nanoparticles in immunogenic cell death: a rational way in cancer immunotherapy

In recent years, ICD has been considered a promising strategy in cancer therapy [391]. The stressed and dying cells release damage-associated molecular patterns (DAMPs) to mediate innate anti-cancer immune response and increase T lymphocyte-induced tumor immunity [392]. The ICD is capable of mediating T cell-induced anti-cancer immune responses against tumor antigens [393]. In recent years, the stimulation of ICD by nanoparticles has been suggested as a promising strategy in cancer immunotherapy. The PLGA nanostructures have been loaded with potassium chloride nanoparticles and then, functionalized with the cancer cell membrane to release K^+^ and CI^−^ ions upon lysosomal degradation. Then, these ions cause cancer cell death by mediating a hypertonic state in which cells secrete adenosine triphosphate (ATP) and high mobility group box 1 (HMGB-1) for stimulation of ICD [394]. The β-D-Glucose-reduced silver nanostructures can release calreticulin and increase the release of HSP70, HSP90, HMGGB1, and ATP [395]. These factors are prerequisites for the induction of ICD. In a novel strategy, the chitosan-coated PLGA nanoparticles have been loaded with HPV16 E7_44 − 62_ peptides and then, their functionalization with ICD tumor cell membrane has been performed to enhance the maturation of dendritic cells for cancer immunotherapy [396]. Furthermore, the Fe_3_O_4_ nanostructures have been modified with living bacteria and cancer cell membranes to cause ferroptosis and ICD for enhancing anti-cancer immune responses [397]. Therefore, the increasing evidence highlight the application of nanostructures for the stimulation of ICD in cancer immunotherapy [304, 398–403]. In an effort, albumin nanostructures have been developed in which IR780, as photosensitizer was loaded in core and cGAS-STING agonists/H2S producer-ZnS was loaded in shell to mediate photodynamic and photothermal therapy. These nanostructures mediated pyroptosis through the caspase-3/GSDME axis in mediating dendritic cell maturation. Then, T cells are activated and improve the potential PD-L1 blockade in cancer immunotherapy [404]. For stimulation of ICD, various kinds of nanoparticles including polymeric nanostructures [405, 406], liposome-modified polysopamine structures [407], cRGD-functionalized TPGS nanoparticles [408], iron (II)-cytosine-phosphate-guanine nanoparticles [297] and iron oxide nanostructures [409] have been introduced to enhance cancer immunotherapy. Therefore, nanoparticle-mediated ICD can cause stimulation of dendritic cells to activate T cells in lymph nodes for increasing cancer immunotherapy.

## Cell membrane-coated biomimetic nanostructures

### Cancer cell membrane-functionalized nanoparticles

Biomimetic nanoparticles are characterized by structures whose surfaces are modified or coated with membranes from other cells. The development of biomimetic nanoparticles aims to enhance stealth properties, preventing their identification by the reticuloendothelial system. Biomimetic nanoparticles exhibit good biocompatibility, making them widely applicable in cancer treatment. Recent studies have explored the potential of biomimetic nanoparticles in cancer therapy, demonstrating their versatility when modified with aptamers [410], facilitating chemotherapy through co-delivery of chemotherapy drugs and natural products [411], and showcasing high penetration and targeting features [412]. They have also been utilized for bioimaging and immunotherapy [413, 414], evading the mononuclear phagocyte system [415] and improving blood circulation time [416].

The application of biomimetic nanoparticles in cancer immunotherapy has shown promising results in tumor suppression. In some cases, chemotherapy using biomimetic nanoparticles can activate the immune system. For instance, cancer cell membrane-functionalized phosphorus dendrimer-copper(II) complexes (1G3-Cu) and toyocamycin (Toy)-loaded polymeric nanocarriers with a size of 210 nm can be cleaved in the TME to release cargo and reduce glutathione levels. By causing mitochondrial dysfunction and endoplasmic reticulum stress, these nanocarriers trigger apoptosis and immunogenic cell death. They accelerate the maturation of dendritic cells and increase T lymphocyte infiltration. With the application of PD-L1 antibodies, the nanoparticles can enhance chemotherapy, impair relapse, and prevent the invasion of cancer [417].

Biomimetic nanoparticles also serve as effective carriers for delivering siRNA. Recognizing PD-L1 as an immune evasion factor, its downregulation by siRNA can expedite cancer immunotherapy and prevent T cell exhaustion [418]. These nanoparticles exhibit the capability to effectively suppress cancer progression in vivo, making them promising candidates for the development of cancer vaccines [419].

The field of cancer therapy has undergone a revolutionary transformation with the application of nanogels as drug carriers [420, 421]. Nanogels exhibit favorable physicochemical features. They are potential carriers for delivering both hydrophobic and hydrophilic drugs [422], recombinant proteins [423], and genetic materials [424].

Nanogel-induced immunotherapy has proven effective in impeding cancer progression. Polymeric nanogels, developed from PDEA-co-HP-β-cyclodextrin-co-Pluronic F127 and a charge-reversible polymer named dimethylmaleic anhydride-modified polyethyleneimine, undergo degradation in the TME. These nanogels, functionalized with cancer cell membranes, co-deliver paclitaxel and IL-2, inducing the maturation of dendritic cells and enhancing the infiltration of effector cells [425].

Stimuli-responsive biomimetic nanocarriers have been engineered to optimize cancer immunotherapy. Polydopamine-CaCO_3_ nanocarriers, functionalized with cancer cell membranes and featuring pH-responsive characteristics, enable phototherapy and bioimaging. Exposure to the TME triggers the degradation of nanocarriers, releasing CO_2_ bubbles that promote phototherapy-mediated immunotherapy. Combining this with checkpoint inhibitors further enhances tumor immunotherapy [426].

In the realm of biomimetic nanovaccines, studies have primarily focused on delivering therapeutics or stimulators to dendritic cells. However, the presence of the endocytosis system and endosomal degradation can hinder the effectiveness of these nanovaccines. To address this issue, biomimetic nanoplatforms have been developed to accelerate the internalization of nanoparticles in dendritic cells. Utilizing ROS-responsive nanoparticulate cores fused with peptides and cell membranes, these nanovaccines induce micropinocytosis, facilitating direct cytosolic delivery of Stimulator of Interferon Genes (STING) agonists. This enhances dendritic cell maturation and T cell priming through STING upregulation in cancer immunotherapy [427].

The stimulation of immunogenic cell death and the promotion of dendritic cell maturation and T cell infiltration represents the primary strategy utilized by biomimetic nanocarriers in cancer immunotherapy. Regulation of metabolites is crucial for achieving better immunotherapy responses and immunogenic cell death. Zinc ions-bonded ZIF-8 frameworks with CuS nanodots, functionalized with cancer cell membranes, have been introduced to amplify photothermal-mediated immunotherapy through Zn^2+^ metabolic modulation. These frameworks induce immunogenic cell death, enhance dendritic cell maturation, and increase T cell infiltration [428]. Although less explored compared to macrophage- and cancer-derived membranes, the membranes of red blood cells may also be utilized for the development of biomimetic nanocarriers [429, 430].

One of the main reasons for the modification of nanoparticles with the cancer cell membrane is to provide an antigen resource [431, 432]. The PLGA structures have been functionalized with the membrane of melanoma cells and then, monophosphoryl lipid A (MPLA) as an adjuvant was embedded into nanoparticles to stimulate the maturation of dendritic cells for enhancing antigen-specific T cell response [433]. Since the expression of MHC-I can be found in all cells, such as tumor cells, the cancer cell membrane-functionalized nanostructures can be considered novel APC to induce T cells, even in the absence of professional APCs. The CD80-expressing cancer cells were utilized to derive cell membranes for coating nanoparticles. These nanostructures can directly induce antigen-specific T cells through the interaction of CD28 with cognate T cell receptors, suppressing tumorigenesis in prophylactic and therapeutic tumor models [288]. Furthermore, the cancer cell-membrane functionalized nanoparticles could be considered as vaccines. Despite significant efforts to highlight the potential of cancer cell membrane-functionalized nanoparticles in cancer immunotherapy, there are several limitations to be considered for future studies. Recent studies have highlighted the potential of ferroptosis in cancer immunotherapy [434–436]. More effort regarding the application of biomimetic nanoplatforms in the regulation of ferroptosis and related pathways should be performed to facilitate cancer immunotherapy. Moreover, autophagy is another factor in the regulation of cancer immunotherapy [437–440]. The biomimetic nanoparticles should be designed in a rational way to modulate autophagy for the regulation of T cells and other components of immune systems as well as reprogramming macrophages for effective cancer immunotherapy.

### Red blood cell-functionalized nanoparticles

Red blood cells (RBCs) have obtained much attention for the purpose of drug delivery due to a number of characteristics, including biocompatibility, biodegradability, long lifespan, and desirable encapsulation capacity [441]. The OVA-encapsulated RBCs are able to stimulate CD^4+^ and CD^8+^ T cells after intravenous injection in mice [442]. There is also a promising approach in which RBC membrane is utilized to coat the nanostructures for the development of vaccines with long circulation ability [443]. In an attempt, RBC membrane-functionalized PLGA nanoparticles were designed to deliver both antigen hgp10025 − 33 and adjuvant MPLA. In order to selectively target the dendritic cells, mannose was included in the RBC membrane, and these structures demonstrated high potential in suppression of melanoma [444]. However, the studies are limited, and more experiments regarding the application of other kinds of nanostructures, such as metal and carbon nanomaterials, and their modification with RBC membrane should be performed to improve the potential in cancer therapy.

### Platelet-functionalized nanoparticles

Platelets are released by megakaryocytes, and they can control homeostasis, tumor invasion, and overall physiological and pathological events. Since the platelets have shown expression of self-recognized proteins, including CD47, they are beneficial for reducing clearance and stimulation of the complement system to enhance the blood circulation time of nanostructures [445, 446]. The platelet membrane-functionalized nanoparticles have been exploited to deliver R848 as a TLR7/8 agonist in enhancing accumulation at the tumor region and promoting the interaction with TME components. Moreover, even at low doses (18 µg vaccine per mouse), they could suppress tumors in 87.5% of mice [447]. Moreover, there is the possibility of embedding metformin and IR780 as photosensitizers in platelet membranes to stimulate ICD and improve the potential in cancer immunotherapy through suppressing MDSCs and Treg cells [448]. However, the potential of these nanocarriers in the regulation of TAMs and CAFs for cancer immunotherapy should be highlighted.

### Macrophage membrane-functionalized nanoparticles

The advantage of pH-sensitive biomimetic nanoparticles lies in their ability to induce cancer immunotherapy in a more specific manner due to targeted decomposition in the TME. Immunotherapy with the use of biomimetic nanocarriers may be enhanced through phototherapy. Photo-responsive nanocarriers, through the acceleration of ROS generation, mediate immunotherapy. Macrophage membrane-based vesicles, functioning as nanoparticles, deliver drugs and photosensitizers (TAPP) to induce pyroptosis. These biomimetic vesicles, releasing copper ions, mediate ROS-induced pyroptosis. Utilized as nanoparticles, they increase ROS levels, inducing pyroptosis through the upregulation of caspase-3-induced gasdermin E, resulting in pyroptosis-induced immunotherapy [449].

The macrophage membrane-functionalized nanostructures have shown high potential in specific tumor targeting [450]. The macrophage membrane-coated nanoparticles have been utilized for the delivery of saikopsaponin D, and the surface has been hybridized by adding T7 peptide to provide macrophage-homing capacity for nanostructures and target the tumor cells upregulating transferrin receptor. These nanoparticles specifically accumulate at the tumor site and can escape phagocytosis by the reticuloendothelial system [451]. The functionalization of MOF-derived mesoporous carbon nanostructures with macrophage membranes has been performed to increase their escape from phagocytosis and improve the potential in cancer therapy [452]. In addition to high specificity in tumor targeting, the macrophage membrane-functionalized nanoparticles demonstrate high biocompatibility that, along with their anti-cancer activity, are promising candidates for tumor eradication [251, 453]. Therefore, increasing evidences highlight the application of macrophage membrane-functionalized nanoparticles in cancer therapy [454–459]. In order to better highlight their potential, it is suggested to develop biomimetic nanoparticles functionalized with cancer and macrophage membranes to improve potential in cancer immunotherapy.

**Fig. 5:**
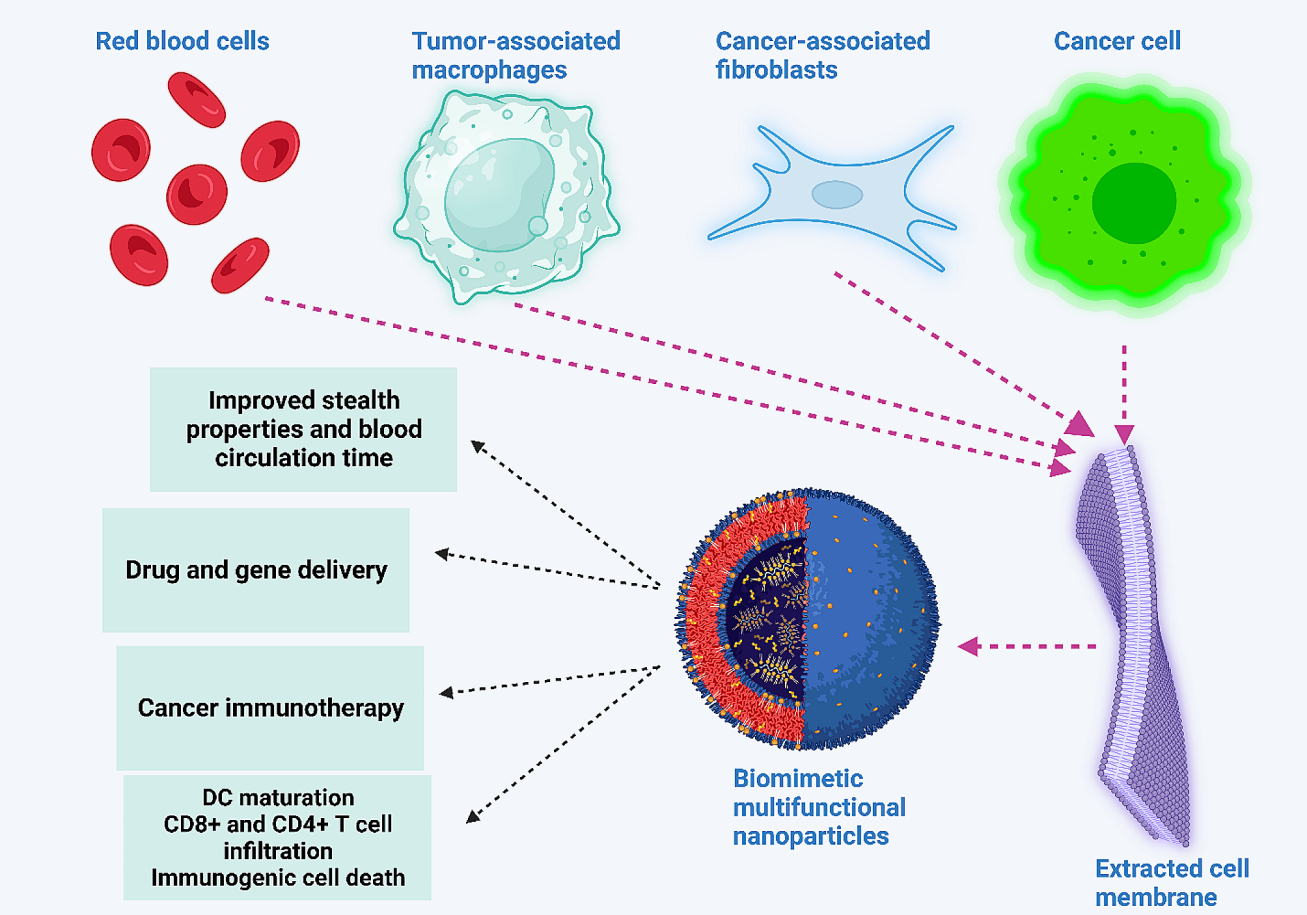
Biomimetic nanoparticles may be developed by the extraction of membranes from red blood cells, cancer cells, TAMs and CAFs. These modifications improve the potential of nanoparticles in cancer immunotherapy. Biomimetic nanoparticles may be utilized for drug and gene delivery by improving stealth properties. They demonstrate prolonged blood circulation and can induce maturation of dendritic cells, increase infiltration of CD^4+^ and CD^8+^ T cells, and cause immunogenic cell death. (Created by Biorender.com)

The biomimetic nanoplatforms are also interesting for the regulation of specific mechanisms such as hypoxia and lipid metabolism in TME. Biomimetic nanoparticles may be designed to present tumor antigens and co-stimulatory molecules simultaneously for cancer immunotherapy [460]. The most effective strategy thus far is centered around the development of biomimetic nanoparticles with pathogen-like features. These biomimetic nanoparticles can elicit significant and long-term immune responses in cancer therapy. One of the key reasons for the application of biomimetic nanoparticles is their ability to enhance the blood circulation time of cargo. Cholesterol reduction in the membrane used for nanoparticle functionalization improves blood circulation time and its effectiveness in inducing cancer immunotherapy [461]. Because of rapid cancer cell proliferation, the hypoxic and nutrient-deficient conditions within the TME hinder the proper functioning of immune cells. The development of a biomimetic platform to increase glucose and glutamine levels required by T cells can reprogram the TME, and accelerate cancer immunotherapy [462]. Table 3; Fig. 5 summarize the application of biomimetic nanoparticles in cancer immunotherapy.

**Table 3:** Application of biomimetic nanostructures for enhancement of cancer immunotherapy

Vehicle	Cancer type/Cell line	Size (nm)/zeta potential (mV)	Highlights	Reference
Biomimetic nanovesicles	Breast cancer/4T1 cells	500 nm	Loading 5-aminolevulinate hydrochloride (HAL) and 3-methyladenine (3MA) into cancer cell-derived microparticles Increasing biosynthesis of PpIX in mitochondria, causing ROS generation after irradiation and increasing mitochondrial dysfunction Suppression of mitophagy PD-L1 downregulation to mediate immunogenic cell death	[463]
Hybrid nanoparticles	Breast cancer/4T1 cells	180 nm/−18.93 mV and − 26.4 mV	Development of hybrid nanoparticles from GTe and modification with cancer cell membrane and bacterial outer membrane GTe functions as a radiosensitizer and the membranes can increase anti-cancer immune responses Increasing ROS generation Stimulation of immunogenic cell death	[464]
Biomimetic nanovaccine	-	-	Functionalization of nanoparticles with cancer cell membrane Co-delivery of CpG and propranolol High accumulation in lymph nodes and enough drug release Increasing dendritic cell maturation and antigen presentation Enhancing CD^8+^ T cell priming and Promoting infiltration of B and NK cells Inhibiting the immunosuppressive TME	[465]
Biomimetic PLGA nanoparticles		147.8 nm/-1.8 mV	Delivery of 2-bromo-palmitate by PLGA nanoparticles to increase its potential in breast cancer therapy Functionalization of nanoparticles with cancer cell membrane Downregulation of PD-1/PD-L1	[466]
Porous silicon@Au nanocarriers	Breast cancer/4T1 cells	Up to 243.30 nm	Functionalization of nanocomposites with cancer cell membrane Stimulation of anti-cancer immune responses and relieving immunosuppressive microenvironment Suppressing the proliferation and invasion of cancers	[467]
AIEgens	Breast cancer/4T1 cells	113.2 nm/-12.8 mV	Modification with dendritic cell-derived membrane Accumulation in lipid droplets of cancer cells The presence of cell membrane allows to accelerate hitchhiking of AIEdots into T cells and stimulates them in cancer immunotherapy	[468]
FePSe_3_ nanosheets	Colon cancer/CT26 cells	+ 28.5, + 24.0, + 37.8, and + 0.2 mV	Modification of nanoparticles with cancer cell membrane Loading anti-PD-1 peptide in the nanoparticles Phototherapy-induced immune responses and tumor ablation Suppression of PD-1/PD-L1 axis to stimulate T cells	[469]

## Exosomes as emerging nanostructures for cancer immunotherapy

Exosomes, ranging in diameter from 30 to 150 nm, are extracellular vesicles secreted by eukaryotic cells [470]. They play a crucial role in intercellular communication by carrying lipids, proteins, and nucleic acids. After their secretion into the extracellular matrix, exosomes can be found in various biological fluids such as amniotic fluid, saliva, urine, and breast milk, among others [471, 472].

In recent years, there has been a significant increase in the application of exosomes in the field of cancer therapy, with a heightened focus on the diverse cargo they can transport. Exosomes exhibit the ability to target various cell types within the body and serve as reliable biomarkers for cancer. Advancements in bioengineering techniques have enabled the effective delivery of cargo using exosomes in cancer treatment. The following subsections delve into the exploration of the potential of endogenous exosomes that are naturally secreted by cells in the body, as well as bioengineered exosomes created in the laboratory for the treatment of cancer.

### Endogenous exosomes

The primary source of exosome secretion includes normal cells, stromal cells, cancer cells, and immune cells. Exosomes secreted by breast cancer cells, for example, play a role in inducing immunotherapy by reprogramming macrophage polarization. These exosomes carry PEDF, promoting M1 macrophage polarization by increasing CD80, IRF5, MCP1, and IL-1β levels, while reducing CD206, Arg, TGM2, and TGF-β levels [473].

Despite the immunotherapeutic potential of exosomes, there are endogenously-secreted exosomes that enhance M2 macrophages, thereby contributing to cancer progression. Inflammation, a risk factor for cancer, can initiate cancer development, and the regulatory functions of macrophages in inflammation can alter the pathogenesis of cancer. Myeloid-derived suppressor cells (MDSCs) in the TME secrete exosomes, transferring miR-93-5p to suppress the STAT3 axis. Enrichment of miR-93-5p in these exosomes, induced by IL-6, leads to MDSC differentiation into M2 macrophages, facilitating colitis-induced cancer development [474].

When macrophages polarize into the M2 phenotype, they release exosomes enriched with apolipoprotein E that reduces MHC-I expression, resulting in decreased immunogenicity and induced immune resistance [475]. Detecting exosomes with immunosuppressive/inductive functions involves recognizing their surface biomarkers. Exosomes positive for PD-L1 are implicated in suppressing immune reactions during cancer progression. PD-L1 + exosomes stimulate CD^8+^ T cell exhaustion, enhancing tumorigenesis during cancer metastasis [476]. The secretion of PD-L1 + exosomes involves intricate molecular pathways, where HMGB1 upregulates RICTOR mRNA levels through miR-200 family downregulation, particularly miR-429. This HMGB1 and RICTOR crosstalk increases PD-L1 generation, promoting the activity of PD-L1 + exosomes in cancer immunotherapy [477].

Although PD-L1 + exosomes have carcinogenic functions, other exosomes can suppress PD-L1 expression. Considering the ability of exosomes to induce cancer immunotherapy, they may be utilized as potential vaccines in the future. Cancer-derived exosomes exhibit superior capabilities in dendritic cell maturation and MHC cross-presentation, compared to cytotoxic T lymphocytes. Furthermore, exosomes can reduce Treg cell numbers in cancer immunotherapy by suppressing PD-L1 expression [478]. This insight suggests that bioengineered exosomes, specifically designed to target dendritic cells, may serve as effective vaccines in cancer therapy. Although the discussion on bioengineered exosomes is reserved for the next section, it is noteworthy that cells can be engineered to secrete exosomes for cancer immunotherapy. For example, the nuclei of cancer cells introduced to M1 macrophages can lead to the secretion of chimeric exosomes, selectively accumulating in lymph nodes and priming T cells to stimulate immune reactions against cancer cells [479]. Therefore, exosomes are potential regulators of the immune system in cancer [480]. Exploring the regulation of exosome secretion and biogenesis from cells opens new avenues for controlling cancer immunotherapy.

### Bioengineered exosomes

Exosomes are used in cancer therapy because they are naturally present in the body, making it less likely for them to be identified as foreign and cleared. Their high biocompatibility allows effective cargo delivery and testing in clinical trials. Dying cancer cell-derived exosomes, modified with MART-1 peptide and CCL22-siRNA, induce T cell-mediated responses and suppress Treg expansion [481]. Bioengineered exosomes can produce persistent immunity against cancer cells, and this paves the way for the development of bio-based vaccines. Bioengineered exosomes, painted with HMGB1, stimulate dendritic cells, enhance immunogenicity, and induce long-term immunity against cancer. These exosomes accumulate in lymphoid tissues and enhance T cell function, inducing long-term immunity and suppressing cancer progression [482]. Another approach involves functionalizing exosomes via CpG DNA to stimulate dendritic cells and enhance antigen presentation, showcasing their co-delivery ability in synergistic cancer immunotherapy [483, 484].

Fusogenic exosomes have been synthesized to address cancer cells escaping the immune system due to self-recognition. These exosomes introduce viral antigens, stimulate dendritic cells, and present antigens to T lymphocytes for CD^8+^ T cell cross-priming [485]. Exosomes from bone marrow-mesenchymal stem cells, loaded with galectin-9 siRNA and oxaliplatin, induce immunogenic cell death, recruit T lymphocytes, reduce Treg cells, and promote M1 polarization of macrophages, contributing to cancer immunotherapy [486]. Engineered M1 macrophage-derived exosomes, targeting TAMs with IL4RPep-1, NF-κB p50 siRNA, and miR-511-3p, induce cancer immunotherapy by reprogramming macrophages and restricting cancer proliferation [487]. Considered as biocompatible carriers, exosomes may be used to modify other nanoparticles. Combining exosomes and liposomes enhances gene delivery, suppresses CD47 signal, and increases CD^8+^ T cell potential. This strategy has the potential for clinical applications in cancer immunotherapy [488]. Table 4 is a summary of the use of exosomes in cancer immunotherapy.

**Table 4:** Application of exosomes in cancer immunotherapy

Exosome source	Cargo	Cancer type	Cell line	Remark	Reference
Dendritic cell	Neoantigens	Melanoma	B16F10 cells	Delivery of cargo to the lymph nodes and stimulation of T- and B-cell immune responses High biocompatibility Improving survival of animal model Suppressing proliferation and delayed tumor relapse	[489]
Glioblastoma	LncRNA	Glioblastoma	Human glioma cell line LN229, mouse glioma cell line GL261, human microglial cell line HMC3, and mouse microglial cell line BV-2	Stimulation of microglia to generate and secrete complement C5 in chemotherapy resistance development	[490]
M1 macrophage	HOTTIP	Head and neck cancer	Hep-2 cells	TLR5/NF-κB overexpression to impair progression of head and neck cancer	[491]
CD45RO- CD^8+^ T cell	-	Endometrial cancer	Ishikawa, RL95-2 and KLE cells	The exosomes suppress estrogen-induced endometrial cancer progression through miR-765 release	[492]
M1 macrophage	SN38 MnO_2_	Breast cancer	4T1 cells	Cancer-targeting ability and prolonging blood circulation Stimulating M1 polarization of macrophages Increasing recruitment of NK cells	[493]
γδ-T cells	-	Nasopharyngeal cancer	NP69, HK-1 and NPC43 cells	Elimination and killing tumor cells Stimulation of FasL and DR5/TRAIL axis Suppressing cancer growth Increasing survival of animal model Apoptosis induction Increasing migration of T cells to the tumor site through CCR5 upregulation	[494]
-	-	Breast cancer	4T1 cells	The smart and bioengineered exosomes with CD62L and OX40L can induce T cells and suppress Treg cell function	[495]
Dendritic cells	-	Melanoma	B16-OVA cells	Functionalization of exosomes with anti-CD3 and anti-EGFR to bind to T cells	[496]
iPSCs and dendritic cells exosomes	Doxorubicin	Gastric cancer	MFC cell line	Delivery of chemotherapy drug Tumor-targeting ability Recruitment of immune cells to the TME	[497]
Cancer cells	Paclitaxel	Breast cancer	4T1 cells	Development of liposome-exosome conjugate with high biocompatibility to increase the number of CD^8+^ T cells	[498]
Cancer cells	-	Breast cancer	4T1 cells	A combination of oxygenated water and cancer-secreted exosomes induce anti-tumor responses and suppress angiogenesis and invasion	[499]
Cancer cells	-	Pancreatic cancer	PANC-1 cells	Exosomes reduce the levels of HLA-DR on the surface of CD14 + monocytes to cause immunosuppression through regulation of STAT3, stimulation of arginase expression and ROS	[500]
M1 macrophages	Docetaxel	Breast cancer	4T1 cells	The docetaxel-loaded exosomes stimulate cancer immunotherapy through M1 polarization of macrophages	[501]
Dendritic cells	siRNA	Melanoma	B16-F10 cells	BRAF siRNA delivery by exosomes to induce T lymphocytes	[502]
HEK 293T cell	Chlorin e6 (Ce6) and immune adjuvant R848	Prostate cancer	RM-1 cells	The exosomes preferentially accumulate in the tumor site and induce dendritic cell maturation Increasing levels of CD80 and CD86 as biomarkers of dendritic cells Inducing M1 polarization of macrophages	[503]

## Stimuli-responsive nanostructures for cancer immunotherapy

### pH-sensitive nanostructures

Smart nanoparticles responsive to TME stimuli may be used to generate targeted delivery systems. Their multifunctionality stems from changing structures in response to TME stimuli, facilitating cargo release at the tumor site. The TME, because of its acidic pH, is distinctive from normal cells, making pH-sensitive nanoparticles ideal for cancer immunotherapy. Nanoparticles have been synthesized using a PLA-b-PEG core and a cytosine (C)-rich i-motif DNA surface to produce the so-called nanovaccines. They are used to deliver cyclic dinucleotides such as cyclic dimeric guanosine monophosphate (CDG) to endosomes, accelerating anti-tumor immunity and preventing TME immunosuppression. In TME’s mild acidic pH, a conformational switch releases cargo, while physiological pH minimally increases CDG release. These pH-sensitive nanoparticles protect CDG from enzymatic degradation, releasing the cargo into the cytoplasm, and stimulating polarization of macrophages into the M1 phenotype [504].

A groundbreaking achievement in cancer immunotherapy involves the creation of prodrug nanoparticles for cargo delivery. These nanoparticles release cargo in response to the pH of the TME. An innovative self-cascaded unimolecular prodrug was designed, comprising an acidic pH-activatable doxorubicin, and an aggregation-induced emission luminogen (AIEgen) photosensitizer linked to a caspase-3-responsive peptide. This engineered prodrug exhibits dual functionality- it can actively release doxorubicin in the acidic TME and initiate apoptosis-related caspase-3 activation. Furthermore, the activated caspase-3 can induce the release and *in-situ* aggregation of photosensitizers. This process enhances dendritic cell maturation, increases CD^8+^ T cell numbers, and prevents metastasis to lung tissue [505].

Mounting evidence supports the combination of chemotherapy and immunotherapy using pH-sensitive nanoparticles for cancer suppression. For example, poly(L-histidine) (PHIS) has been used to encapsulate R848, forming pH-sensitive PHIS/R848 nanocores. Conjugating doxorubicin to hyaluronic acid creates prodrug nanoparticles that coat PHIS/R848. These nanocarriers undergo a switch from hydrophobic to hydrophilic in response to pH change, releasing R848 to modulate the immune system. At pH 5.5, the hydrazone bond cleaves, releasing doxorubicin. The latter is internalized into tumor cells via endocytosis. This activates the immune system and suppresses cancer proliferation [506].

Metal-based nanoparticles can also be designed to decompose in response to TME pH. A human serum albumin-coated zeolite imidazolate framework-8 has been developed for the delivery of a photosensitizer (HPZ), for selective recognition of the TME [507]. This framework enables rapid elevation of zinc ion concentrations while ensuring controlled release of the encapsulated photosensitizer. Under physiological pH (7.4), HPZ exhibits a size of approximately 170 nm, decreasing significantly to less than 10 nm in acidic conditions (pH 6.5). The acid-induced decomposition of HPZ prompts a swift increase in zinc ion concentration, exerting potent cytotoxic effects against colorectal, breast, and pancreatic cancers. Intravenous administration of HPZ in a CT26 tumor-bearing mouse model results in the expansion of T helper and cytotoxic T cells, and reduction in the Treg cell population. These changes lead to a significant inhibition of tumor growth.

While the primary focus of this section is to assess the role of nanoparticles in cancer immunotherapy, it is worth mentioning that microneedle arrays, composed of tiny needles, can also be used for sustained cargo delivery. pH-sensitive nanoparticles can be incorporated into microneedles, facilitating sustained release for hyperactivating the immune system and creating nanovaccines [508]. Hydrogels are also available for sustained cargo delivery. For example, nanoparticles can be embedded in pH-sensitive hydrogels for delivering doxorubicin and JQ1 (a small molecular inhibitor) to induce immunogenic cell death [509]. Of note, pH-sensitive biomimetic nanocarriers, crafted from PLGA and coated with membranes from macrophages and cancer cells, can deliver FGL1-siRNA and metformin for cancer immunotherapy. These biomimetic nanoparticles enhance cargo endosomal escape, with metformin suppressing PD-L1 through AMPK upregulation, and siRNA reducing FGL1 expression to boost anti-cancer immunity through T cell induction [510]. Given the impact of TME pH on tumorigenesis, pH regulators have been developed. Calcium carbonate nanostructures neutralize TME pH by consuming lactate, promoting M1 macrophage polarization, immune cell infiltration, dendritic cell stimulation, and T cell recruitment for cancer immunotherapy [511]. Table 5 summarizes the application of pH-sensitive nanoparticles in cancer immunotherapy.

**Table 5:** pH-sensitive nanoparticles in cancer immunotherapy

Nanoparticle	Cancer type/cell line	Size (nm)/zeta potential (mV)	Highlights	Reference
PEG/PEI/CAD nanoparticles	Breast cancer/4T1 cells	At a range of 100–250 nm/at a range of 10–20 mV	Delivery of doxorubicin and its release in a pH-sensitive manner Immunogenic cell death induction The acidity of the endosome induces cleavage of *cis*-aconityl Recruitment of dendritic cells	[512]
Hollow silica nanostructures	Breast cancer/4T1 cells	100 nm/+11 mV	Increased retention in response to low pH level of TME Targeting mitochondria and increasing ROS levels Stimulation of photodynamic therapy Combination with checkpoint inhibitors mediates anti-tumor immunity	[513]
Dextran-modified BLZ-945 nanocarriers	Breast cancer/4T1 cells	11.35, 112.4, and ∼135.6 nm	Presence of a borate ester bond as a pH-sensitive bond Immunogenic cell death induction Dendritic cell maturation, TAM depletion and T cell infiltration	[514]
Manganese nanoparticles	Melanoma/B16-OVA cells	130 nm	Mn2 + and 2-methylimidazole (2-MI) have been used to encapsulate ovalbumin with pH-sensitive features and the ability of dendritic cell maturation in cancer immunotherapy	[515]
Mesoporous silica nanostructures	-	146 nm	pH-sensitive feature and delivery of R848 Uptake of nanoparticles by antigen-presenting cells Stimulation of dendritic cells and boosting T cell-mediated immune responses	[516]
Peptide-functionalized nanobubbles	Breast cancer/4T1 cells	173.8 nm/-1.53 mV	Functionalization of nanobubbles with anti-PD-L1 peptide Loading Ce6, metformin and perfluorohexane in nanobubbles Accumulation of nanoparticles in acidic pH causes detachment of PEG ligands and then, exposure of peptide to suppress PD-L1 Hypoxia relief by metformin and increasing potential of Ce6 in cancer therapy Increasing anti-tumor immunity and prevention of immunosuppression	[517]
Polymer-lipid complexes	Lymphoma/E.G7-OVA cells	-	The polymer-lipid-embedded liposomes release ovalbumin in response to pH and stimulate anti-cancer immunity by releasing ovalbumin in the cytoplasm of dendritic cells	[518]
Polymer-modified liposomes	Lymphoma/E.G7-OVA cells	100 nm/−15.7 mV and 1.3 mV at pH 7.4 and pH 5.5	pH-responsive feature and cationic lipid inclusion Delivery of ovalbumin Increasing cytokine generation Antigen presentation through MHC-I and MHC-II	[519]
Liposome	Lymphoma/E.G7-OVA cells	136, 108 and 115 nm/-0.87, -11 and − 6.1 mV	Modification of liposomes with polymer Destabilization of liposomes in pH 6 Uptake of liposomes by dendritic cells Delivery of ovalbumin to cytosol Tumor growth suppression	[520]
Polymer-modified liposomes	Lymphoma/E.G7-OVA cells	97, 100, 88, 110, 108 and 109 nm/-18, -19, -11, -63, -65 and − 60 mV	Inclusion of cationic lipid and CpG-DNA Inducing dendritic cells to secrete cytokines Stimulation of antigen-specific immune responses pH-sensitive feature	[521]
Biomimetic nanoparticles	Breast cancer/4T1 cells	102.86 nm	Coating manganese nanoparticles with hybrid membranes Membrane is developed from mesenchymal stem cell membrane and pH-sensitive liposomes Targeted delivery of BPTES Inducing STNG pathway and M1 polarization of macrophages Relief of immunosuppression TME	[522]
Polysaccharide-based polymers	Lymphoma/E.G7-OVA cells	157 nm/-50 mV	Stimulation of dendritic cells Cytoplasmic delivery of antigen Th1 cytokine production by dendritic cells	[523]
Liposomes	Melanoma/B16-OVA cells	401, 754, 636 and 674 nm	pH-sensitive liposomes deliver STING and TLR9 agonist Increasing Th1 immune responses in tumor suppression	[524]

### Redox-sensitive nanoparticles

Redox-sensitive nanoparticles are carriers that release cargo in response to redox imbalances in the TME. The development of these nanoparticles involves introducing redox-sensitive bonds, such as disulfide bonds. They have found application in cancer immunotherapy, capable of stimulating various immune cells.

Multifunctional nanocarriers with redox-sensitive features, comprising toll-like receptor agonists, catalase, and PD-L1-siRNA, have been designed for cancer therapy. These nanoparticles induce M1 polarization of macrophages, increase ROS production, downregulate PD-L1 expression, and enhance CD^8+^ T cell functions by suppressing Treg cells [525].

Clinical application of immunotherapy hinges on using biocompatible and long-term safe nanostructures like micelles. Micelles containing doxorubicin and R848, a TLR7/8 agonist, were experimentally used as nanovaccines. Elevated glutathione levels in the TME trigger micelle decomposition, releasing cargo and inducing immunogenic cell death, dendritic cell stimulation, and accelerated immune responses. Additionally, redox-responsive polymers and an A2AR antagonist within micelles suppress adenosinergic signaling to activate NK and CD^8+^ T cells in cancer immunotherapy [526].

Cationic polymer dots, known for their small particle size, imaging capabilities, and drug delivery potential, have seen extensive use in biomedicine. PEI600-modified redox-sensitive hyperbranched poly(amido amine) nanostructures, partially carbonized with polymer dots, were used for carrying ovalbumin. These structures enhance splenocyte proliferation, elevate cytokine levels (including IL-12 and IFN-γ), promote dendritic cell maturation, and increase CD^4+^ and CD^8+^ T cell counts, as well as T lymphocytes. Ovalbumin release from these structures is redox-responsive [527].

Given that both pH and redox serve as endogenous stimuli, new nanocarriers have been developed that exhibit dual responsiveness. pH- and redox-sensitive micelles containing ovalbumin, modified with PLH-PEG, are used as cancer vaccines. These micellar nanostructures enable cytosolic delivery of ovalbumin through redox release, proton influx, micelle disassembly, and ultimately, a proton sponge effect and lysosome break. These micelles enhance MHC-I rates, antigen presentation, lymph node accumulation, and improve immune reactions [528].

### Photo-responsive nanoparticles and phototherapy

Light serves as an exogenous stimulus in nanoparticle development, where laser irradiation induces bond cleavage to release the loaded cargo. This forms the basis of photodynamic therapy (PDT) and photothermal therapy (PTT) for cancer ablation. In PDT, ROS production induces cell death, while PTT causes cell death through hyperthermia. Combining phototherapy with immunotherapy can enhance cancer suppression. Two methods are utilized, including a combination of phototherapy and immunotherapy or stimulation of phototherapy-mediated immunotherapy. Both methods offer a high likelihood of tumor suppression, improving the fight against cancer. PLGA nanoparticles loaded with R837, docetaxel, and PB agents are used for immunotherapy and PTT. These nanoparticles are modified with cancer cell membranes for enhanced effectiveness. R837 stimulates dendritic cell maturation, and docetaxel increases M1 polarization of macrophages, impairing the immunosuppressive TME. This leads to increased infiltration of cytotoxic T lymphocytes in the TME for effective cancer immunotherapy [529].

Similar to doxorubicin, which induces immunogenic cell death, docetaxel has shown potential in cancer immunotherapy by regulating TAM polarization. CuS nanoparticles with NH_2_ functional groups, functionalized with folic acid and conjugated to PEI-PpIX for enhanced solubility, deliver docetaxel and CpG, inducing PTT and immunotherapy. These nanoparticles exhibit excellent photothermal conversion ability upon exposure to 650 and 808 nm laser irradiation and induce M1 polarization of macrophages through the function of docetaxel in cancer immunotherapy [530].

Beyond combining immunotherapy and PTT, it is possible to induce immune reactions resulting from PTT. Tumor cell killing and antigen release can accelerate immune reactions. A peptide-photosensitizer conjugate, developed from anti-PD-L1 peptide, cleavable by MMP-2, and purpurin 18 as a photosensitizer, accumulates in the tumor site. MMP-2 enzyme degradation releases the peptide, causing antigen release. Irradiation induces dendritic cell maturation, migrating into lymph nodes, increasing T cell infiltration, suppressing metastasis to lung tissue, and eliciting anti-tumor immune responses [531].

Photo-responsive structures can also function as vaccines. Chelation of Fe^3+^ ions with ovalbumin leads to biomineralization into nanovaccines, embedding IR820 as a photosensitizer through electrostatic incorporation. The presence of iron induces ferroptosis, mediating immunogenic cell death. Immunogenic cell death stimulates neoantigens and DAMPs, synergizing with ovalbumin in cancer immunotherapy. Near-infrared irradiation induces PTT, enhancing immunotherapy. These nanostructures enhance T cell infiltration, inhibit the primary tumor, and show promising impacts in combination with checkpoint inhibitors [532].

The combination of PDT and immunotherapy has shown promising results in cancer immunotherapy. Although PDT and PTT have different mechanisms of action, both can be combined with immunotherapy to expedite the immunotherapeutic process by exposing antigens or inducing cell death to stimulate immune responses. Photodynamic therapy is preferred over PTT due to potential hyperthermia effects on normal tissues. However, when tumor-targeted nanoparticles are developed, they can precisely execute PDT in the tumor site, effectively killing cancer cells. Various nanoenzymes with cancer cell accumulation can be developed to induce PDT and enhance cancer immunotherapy.

A nanoparticle core was created by connecting paclitaxel drugs through a disulfide bond (PTX-SS-PTX), with P18 as a photosensitizer connected to MPEG-CPPA-b-P(M4). In response to glutathione, the disulfide bond degrades, and laser irradiation in response to ROS increases singlet oxygen generation, releasing high mobility group box 1 (HMGB1) due to GSDME activation. HMGB1 releases and pyroptosis induction leads to dendritic cell maturation, educating naïve T cells in lymph nodes, expanding T cells, and developing memory T cells for cancer immunotherapy [533].

For PDT stimulation, the photosensitizer is loaded into nanoparticles. Recent studies have shown the potential of herbal medicine in cancer treatment. Mesoporous silica nanoparticles modified with PEG can carry both chlorin e6 and astragaloside III, stimulating NK cells and suppressing cancer cell growth. These nanoparticles accumulate in the tumor site, increasing immune cell infiltration, promoting the activity and cytotoxicity of CD^8+^ T cells and NK cells, and impairing tumorigenesis [534].

Liposomes are also potential nanocarriers in cancer immunotherapy. Liposomes carrying IPI-549 as a PI3Kγ inhibitor and chlorin e6 as a photosensitizer induce ROS generation after irradiation, facilitating immunogenic cell death and improving the potential of T lymphocytes in eliminating cancer cells. IPI-549 delivery by nanoparticles can decrease arginase-1 (Arg-1) and ROS levels, increasing apoptosis in MDSCs, and preventing their suppressive function on T cells. In addition, these nanoparticles decrease the infiltration of M2-polarized macrophages and mature dendritic cells in cancer immunotherapy [535].

**Fig. 6:**
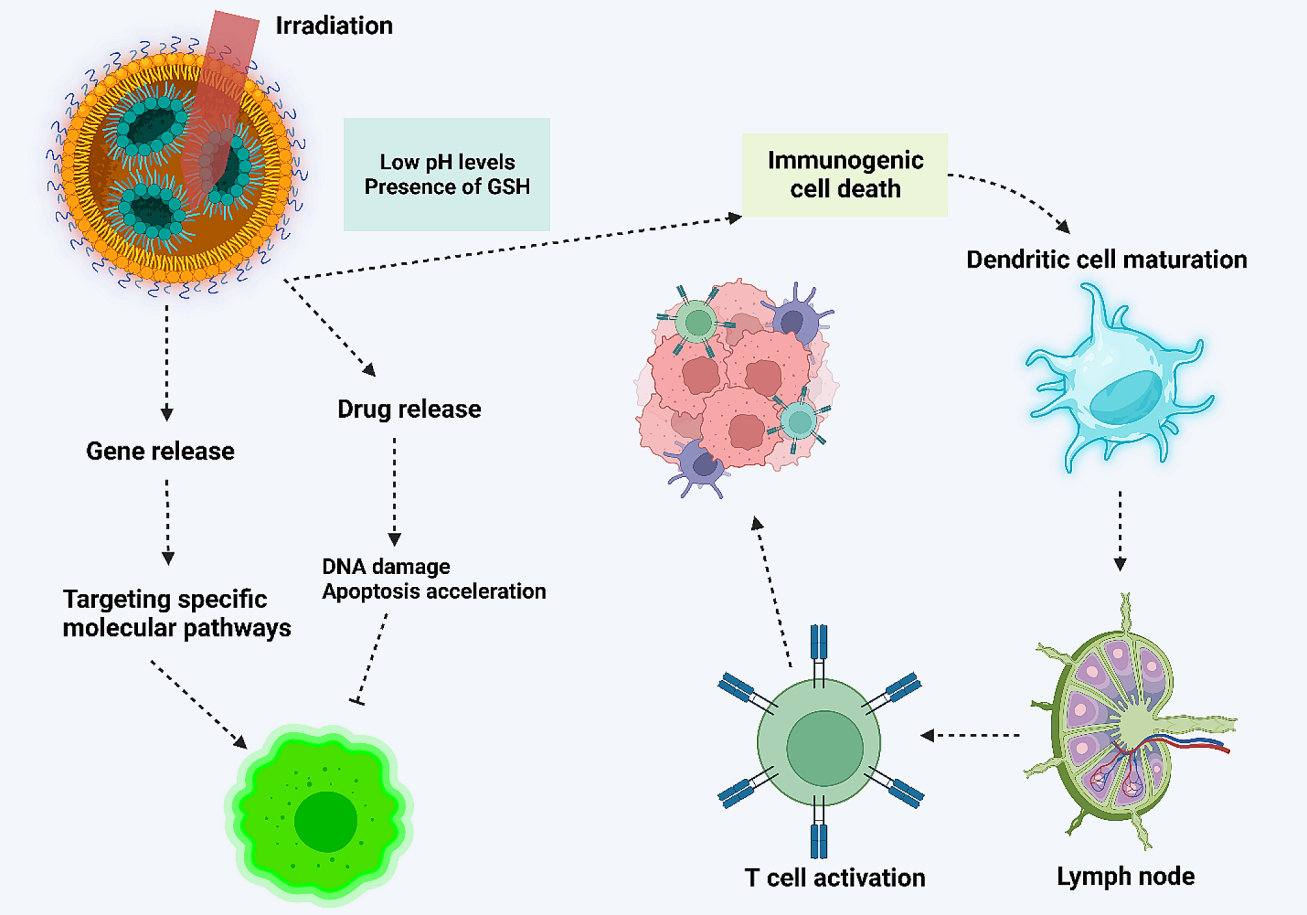
Stimuli-responsive nanocarriers in cancer immunotherapy. Nanoparticles that respond to pH, redox, or light can release cargo to induce apoptosis, DNA damage, and regulation of molecular pathways. Moreover, stimuli-responsive nanocarriers stimulate immunogenic cell death to increase dendritic cell maturation. They migrate into lymph nodes and increase activation of T cells

In both nanoparticle-mediated PDT and PTT, the most prominent mechanism is the stimulation of immunogenic cell death to accelerate cancer immunotherapy. Increasing evidence highlights the application of nanoparticle-mediated phototherapy and immunotherapy in cancer suppression [536–540]. Table 6 summarizes the nanoparticles causing PDT and PTT, and their combination and relationship with immunotherapy. Figure 6 shows the application of stimuli-responsive nanocarriers in cancer immunotherapy. Since the pre-clinical studies demonstrate the function of nanoparticles for cancer immunotherapy and tumor suppression, the clinical application of nanostructures in cancer immunotherapy has been performed to evaluate their potential [541]. Table 7 and table 8 summarize the clinical application of nanoparticles in cancer immunotherapy.

**Table 6:** Nanoparticle-mediated PDT and PTT and their relationship with cancer immunotherapy

Nanoparticle	Cancer type/Cell line	Size (nm)/zeta potential (mV)	Outcome	Reference
Nano-PROTACs	Breast cancer/4T1 cells	40 and 80 nm/	The nanoparticles have been comprised of PpIX as photosensitizer and SHP2-targeting PROTAC peptide (aPRO) The stimulation of aPRO occurs as a response to upregulation of caspase-3 Targeted degradation of SHP2 through ubiquitin-proteasome system SHP2 depletion suppresses immunosuppressive pathways, including CD47/SIRPα and PD-1/PD-L1, to improve anti-cancer functions of macrophages and T cells	[542]
MRC nanoparticles	Breast cancer/4T1 cells	38.69 ± 0.20	Co-delivery of RGX-104 as an immune agonist and chlorin e6 Stimulation of ApoE by RGX-104 to impair the function of MDSCs and accelerate pyroptosis Chlorin e6-induced PDT to facilitate oxidative damage and enhance immunogenicity	[543]
Ru(II)-modified TiO_2_ nanocarriers	4NQO-Oral cancer	40 nm/ −7.41 ± 1.22 and + 27.65 ± 2.46 mV	Loading HIF-1α-siRNA in nanoparticles Stimulation of PDT and inducing lysosomal damage Downregulation of HIF-1α and enhancing killing of oral cancer Stimulation of CD^4+^ and CD^8+^ T cells	[544]
PDA-FA nanoparticles	Colon cancer/CT26 cells	130 nm/-14.29 mV	Delivery of CpG as immunomodulatory to induce dendritic cell maturation and increase T cell activity Suppressing Treg cells and MDSCs PTT induction	[545]
Copper sulphide nanoplatforms	Melanoma/B16F10 cells	28 nm/30.5 mV	Delivery of Cas9 ribonucleoprotein to target PTPN2 Downregulation of PTPN2 to increase infiltration of CD^8+^ T cells Increasing levels of IFN-γ and TNF-α Improving immune-sensitivity	[546]
Polymer nanoadjuvants	Breast cancer/4T1 cells	40 nm/-31 mV	Doping with TLR agonist as an immunomodulatory adjuvant Presence of lipid shell response to temperature The PTT potential in response to NIR-II Immunogenic cell death induction and release of TLR agonist Upregulation of TLR7/TLR8 and stimulation of immunogenic cell death enhance dendritic cell maturation and amplification of anti-cancer immune responses	[547]
Nanoenzymes	Breast cancer/4T1 cells	100 nm	Cu-doped MoOx (CMO) nanozyme comprises the core that is coated with cancer cell membrane Increasing the tumor accumulation and nanozymes causes oxidative damage through increasing ROS generation PTT causes immunogenic cell death to activate the immune system	[548]
Gold nanorod	Colon cancer/CT26 cells	66.48 ± 1.41, 76.73 ± 4.6, 93.72 ± 2.7, and 116.8 ± 6.5 nm/26 mV	The 808 nm laser irradiation causes PTT Stimulation of immune cells in the lymph nodes	[549]
AIE	Breast cancer/4T1 cells	110.3 nm/+10.68 mV	Modification with cancer cell membrane Stimulation of immunogenic cell death Increasing ROS generation through PDT	[550]
Polymer nanoagonist	Breast cancer/4T1 cells	42 and 50 nm/-19.9 mV	Stimulation of PTT Increasing immunotherapy and induction of immunogenic cell death	[551]
Antigen-capturing nanoparticles	Breast cancer/4T1 cells	41.1 nm	Stimulation of phototherapy under NIR irradiation Increasing antigen uptake and presentation Suppressing cancer progression	[552]
Black phosphorus quantum dot nanovesicles	Breast cancer/4T1 cells	120 nm/-23 mV	Loading them into thermosensitive hydrogels NIR irradiation increases dendritic cell activation and then, they migrate into lymph nodes for the stimulation of CD^8+^ T cells	[553]
Gold nanocages	Colon cancer/CT26 cells	52 ± 3 nm/ -24 ± 2 mV	Delivery of anti-PD-L1 and galunisertib by nanocages Stimulation of PTT to cause immunotherapy	[554]

**Table 7:** The application of nanoparticle-based immunotherapy in clinical studies [541, 555–557]

Nanoparticle	Phase	Remark	Reference
RNA-lipoplexes	Phase I	Increasing maturation of dendritic cells and increasing T cell response	[558]
miR-34a-loaded liposomes	Phase I	Reduction in the expression of immune evasion genes	[559]
miR-4157-loaded lipids	Phase I	Stimulation of neoantigen-specific T cells and increasing anti-cancer immune responses	[560]
Iron oxide nanostructures	Not applicable	Increasing M1 polarization of macrophages from M2 phenotype	[256]
Paclitaxel-loaded lipid core nanostructures	Phase II	Enhancing dendritic cell maturation	[561, 562]
Doxorubicin-loaded anti-EGFR immunoliposomes	Phase II	Stimulation of immunogenic cell death Suppressing EGFR-induced growth signaling	NCT02833766
Plasmid DNA complex-loaded cationic liposomes	Phase I	Stimulation of the immune system	NCT00860522
Combination of anti-PD-1 and hafnium oxide nanostructures	Phase I	Increase in tumor cell death, promotion of immunogenic cell death, and induction of the immune system	NCT03589339 [563]

**Table 8:** The challenges regarding the application of nanoparticles in cancer immunotherapy

Nanoparticles	Benefits	Challenges
Polymeric nanoparticles	• Targeted delivery of cargo to improve the therapeutic index and reduce the systemic side effects • Prolonged release of drugs • Potential in the delivery of various cargoes including small molecule drugs, proteins, peptides, and nucleic acids • Increased stability of drugs and preventing degradation • Stimulation of the immune system • Biocompatibility and biodegradability	• The development of nanoparticles with desirable size, charge, and targeting capacity is challenging • Strict rules regarding clinical application • Unexpected interactions with the immune system • Challenges in the scale-up generation, storage and stability
Lipid nanoparticles	• Efficient delivery of genetic tools including mRNA, siRNA, and DNA • Targeted delivery • Protection of cargo • Long-term biocompatibility and safety • Adjuvant impact that a number of lipid components can function as adjuvants and increase anti-cancer immune responses	• Complex manufacturer production, especially the development of nanoparticles for gene delivery • They require ultra-low temperatures to preserve their stability • Immunogenicity that can lead to inflammation and other side effects • Low loading capacity
Metal nanoparticles	• Targeted delivery of drugs and high loading and encapsulation efficiencies • Application for photothermal therapy, since a number of nanostructures such as gold nanocarriers can absorb light and cause photothermal-mediated tumor ablation • Delivery of immunomodulatory agents for cancer immunotherapy • Synergistic therapy through a combination of drug delivery and photothermal therapy • Imaging and biosensing	• The biodistribution of metal nanostructures is challenging along with their clearance from the body • The metal nanostructures possess high cytotoxicity and poor biocompatibility • The chance of inflammation and immune reactions • Stability and toxicity towards normal cells
Carbon nanoparticles	• High drug-loading potential for the delivery of drugs, proteins, and genetic tools • Application in photothermal and photodynamic therapy • Imaging and biosensing of cancer biomarkers	• The toxicity and poor biocompatibility • The changes in the biodegradation of carbon nanoparticles, leading to their long-term accumulation • Complexity in the generation of nanoparticles at a large scale and achieving the desirable physicochemical properties including size, zeta potential and others • Heterogeneous biological functions among the various classes of carbon nanomaterials including tubes, dots and sheets

## An overview of various classes of nanoparticles in cancer immunotherapy

### Polymeric nanoparticles

Polymeric nanoparticles are among the most commonly applied structures in cancer immunotherapy owing to their biocompatibility, biodegradability, chemical stability, water solubility, and high drug loading [564, 565]. The PLGA, PGA, PLG, PEG, PEI and chitosan nanoparticles have been widely used in cancer immunotherapy [566]. Moreover, such nanostructures have shown potential as immunostimulatory adjuvants in cancer immunotherapy [567–569]. Loading TLR7/8 agonists in PLGA nanostructures enhanced levels of CD40, CD80, and CD86 through stimulation of bone marrow-derived dendritic cells and subcutaneous administration of such nanoparticles stimulated dendritic cells and CD^8+^ T cells [570].

### Lipid nanoparticles

Liposomes are among the promising nanocarriers for drugs, genes and vaccines [571]. Until now, multiple kinds of liposomal nanostructures including 1,2-dioleoyl-3-trimethylammonium-propane (DOTAP), 3β- (N- [N’, N’-dimethyl aminoethane] - carbamoyl) cholesterol (DC-Chol), and dimethyl diocta decylammonium (DDA) [572, 573] have been significantly applied for the antigen exposure to APCs and providing vaccine adjuvants to increase antigen-specific immune reactions [574, 575]. The dextran-functionalized liposomes with pH-sensitivity activity have shown high uptake by dendritic cells and they can provide cytosol delivery of ovalbumin to accelerate antigen-specific immune reactions and impair cancer progression [576]. Furthermore, loading CpG-ODNs as TLR9 agonist and 3,5-didodecyloxybenzamidine as an adjuvant into liposomes can enhance dendritic cell-mediated cytokine production to enhance antigen-specific immunity [521]. Micelles are among another kind of lipid nanoparticles that can function as delivery systems for antigen/adjuvant to improve potential of vaccines. The polymeric hybrid micelles have been utilized for the delivery of CpG-ODN and Trp2 to create a nanovaccine for targeting lymph nodes and enhancing the accumulation of cargo in dendritic cells, triggering CD^8+^ T cell-mediated immune responses and enhancing cancer suppression (melanoma) [577].

### Carbon nanoparticles

Carbon nanotubes (CNTs) have been shown to mediate immunostimulatory impacts in vitro and in vivo. The oxidized multiwalled carbon nanotubes (MWCNT) have shown the ability for the delivery of cancer-testis antigen, known as NY-ESO-1 and CpG-ODNs. These structures showed uptake by dendritic cells and mediated powerful CD^4+^ and CD^8+^ T cell-driven immune reactions to impair melanoma progression [578]. Furthermore, the co-delivery of ovalbumin and CpG-ODN, as well as anti-CD40 Ig as immunoadjuvants by MWCNTs has shown potential in accelerating T cell responses and suppressing melanoma progression [579].,

### Metal nanoparticles

Gold nanostructures are promising factors in cancer immunotherapy owing to their characteristics, including biocompatibility, adjustable surface chemistry, and ease of controlling size and shape [580]. The gold nanostructures have shown potential in inducing differentiation of macrophages into dendritic-like cells to enhance T cell proliferation and promote cytokine release [581]. Furthermore, the gold nanostructures have shown incredible potential to act as adjuvants for enhancing antibody generation [582]. The potential of gold nanoparticles in cancer therapy via TME modulation has been revealed [583, 584]. The surface of hollow gold nanostructures has been functionalized with CpG-ODNs to enhance their cellular uptake and enhance function in the induction of immune responses, including enhancing TNF-α secretion [585]. The silica nanoparticles have also been applied widely in biomedicine for imaging [586], specific targeting of cancer [587], and delivery of drugs and genes [588]. The mesoporous silica nanoparticles have been applied for antigen delivery and acted as vaccines to induce humoral- and cell-driven immune reactions while having high biocompatibility and lacking toxicity [589]. The hollow mesoporous silica nanoparticles have been shown to be biodegradable and are capable of TME remodeling in cancer immunotherapy [361]. According to these studies, various categories of nanostructures demonstrate promising characteristics in cancer immunotherapy, and all of them have shown potential in TME remodeling. The next step for the clinical application of these nanocarriers depends on their biocompatibility and long-term safety that lipid nanoparticles are at the front line.

## Conclusion and challenges

Cancer immunotherapy and the development of vaccines have ushered in a new era in cancer treatment, instilling hope in patients and significantly enhancing prognosis and survival rates. However, challenges persist in the utilization of adjuvants and immunomodulatory agents. This prompted the exploration of novel solutions, particularly through the application of nanoparticles. The intricate interactions within the TME play a pivotal role in influencing cancer cell responses to immunotherapy. In this context, nanoparticles designed for targeted delivery of immunomodulatory compounds and TME remodeling have emerged as promising tools in cancer immunotherapy.

Nanoparticles address the current challenges in cancer immunotherapy by demonstrating the capability to regulate TME components, altering the trajectory of tumor cell progression, and mitigating the immunosuppressive milieu in cancer. Nanostructures designed for TME remodeling can increase the infiltration of cytotoxic T cells, stimulate dendritic cells, induce M1 macrophage polarization, and inhibit MDSCs. Furthermore, nanoparticles serve as effective carriers for delivering adjuvants and other immunomodulatory compounds, such as drugs or genes, in cancer immunotherapy.

A significant breakthrough in nanoparticle development for cancer immunotherapy lies in the creation of biomimetic nanocarriers, incorporating functionalized membranes derived from red blood cells, tumor-associated macrophages, cancer-associated fibroblasts, and tumor cells. In addition, the utilization of exosomes, whether naturally secreted by cells or bioengineered in the laboratory, holds promise in regulating the immune system in cancer therapy. Nanoparticles exhibit the capacity to diminish the number of Treg cells and MDSCs, preventing immunosuppression. Moreover, nanoparticle-induced immunogenic cell death promotes the activation of dendritic cells, facilitating their migration into lymph nodes to stimulate T cells for cancer immunotherapy.

Stimuli-responsive nanocarriers, particularly pH- and redox-sensitive nanoparticles, enhance the potential of cancer immunotherapy. Photo-responsive nanoparticles, through photothermal and photodynamic therapy, contribute to the augmentation of cancer immunotherapy by inducing immunogenic cell death, regulating dendritic cells and T cells, and promoting M1 polarization of macrophages. This multifaceted approach highlights the versatility and promise of nanoparticles in advancing cancer immunotherapy.

The clinical application of nanoparticles involves several crucial considerations [590]. Once we comprehend the potential of nanoparticles in cancer immunotherapy, translation of these technologies, advancements, and findings to the clinical setting becomes imperative. Clinical trials involving immunotherapy for cancer patients are already underway. In the context of solid tumors and their clinical treatment, leveraging nanoparticle-induced phototherapy is recommended to enhance immune responses and stimulate dendritic cells along with their maturation.

Last, but not least, it is essential to assess the biocompatibility of nanoparticle-induced immune responses to prevent systemic immune reactions against normal cells. Evaluation of delivering immunomodulatory agents alongside chemotherapy drugs should be conducted to determine the synergistic impact on cancer therapy. However, careful consideration must be given to whether the associated side effects are tolerable for patients. Therefore, assessing the biocompatibility and toxicity profile of nanoparticles stands out as a critical aspect of their clinical application.

Currently, a high number of studies have focused on the clinical utilization of nanostructures for boosting immunotherapy [591]. One of the most prominent drawbacks is that currently developed nanoplatforms are in phase I or II. The present review provided the possible interaction and function of nanoparticles with TME components and immune cells to induce cancer immunotherapy that can be used in clinics. In most cases, animal models have been utilized to evaluate the potential of nanoparticles in cancer immunotherapy, making it hard to translate to humans. The selection of humanized animal models can improve the chance of translation into the clinic. Moreover, the appropriate nanoparticles should be chosen for cancer immunotherapy. In this way, it is suggested to use FDA-approved agents such as polymers or lipid nanoparticles to accelerate the pace of clinical translation and immune regulation. The biosafety, tolerability, and reproducibility of nanocarriers should also be considered for clinical application. In recent years, a high number of nanoparticles have been introduced into clinics for the treatment of cancers, including lipid-based nanoparticles (generic name: JVRS-100) for the treatment of leukemia (NCT00860522), liposomes for the treatment of lymphoma, melanoma, breast, ovarian and prostate cancers (NCT03349450, NCT01052142, NCT01095848), PLGA nanoparticles for the treatment of metastatic melanoma (NCT01753089) and colloidal gold structures for the treatment of advanced solid tumors [592]. However, all of these nanoparticles have been evaluated in phase I and II studies.

Taking everything together, there are a number of points that should be considered for the application of nanoparticles in cancer immunotherapy [593]. Currently, cancer immunotherapy is mainly based on the application of nano-scale delivery systems for conventional immunotherapeutic compounds, including antibodies, recombinant proteins, and small molecular-based immune agonist, adjuvant, or inhibitor. The rationale for the application of nanoparticles is to improve the pharmacokinetic profile of immunotherapeutic agents, diminish the side impacts, prevent the cytokine storm by immune hyperactivation, and ameliorate deficiency in immune responses. Moreover, nanoparticles are promising for reversing immunosuppression and preventing immune evasion. Although this is an interdisciplinary field combining biology and engineering, a number of factors should be carefully investigated, including nanoparticle-mediated immunogenicity, biocompatibility and engineering aspects regarding therapeutic compound loading in nanostructures. Moreover, several nanoparticles have anti-cancer activity, causing synergistic impact with immunotherapeutics. One of the most important features of nanoparticles is their ability for controlled release of therapeutics. The hyperactivation of immune systems using immune agonists or adjuvants can affect normal cells and tissues. Therefore, the delivery of therapeutics should be performed in a controlled manner and safe levels should be delivered. Moreover, the rapid or burst release of immunotherapy compounds is not effective in providing long-term anti-cancer immunity. A number of nanoparticles are promising for the controlled release of therapeutic compounds, including PLGA nanostructures that, upon degradation of the polymer, the release of the immunotherapeutic compound occurs [594, 595]. Biocompatibility has been another important factor in pre-clinical and clinical studies [596]. Along with controlling the toxicity of immunomodulatory factors, including recombinant cytokines [597–599], the physicochemical characteristics of nanostructures including size, shape and thermal conversion ability, among others, should be adjusted in a manner to reduce the side effects of nanoparticles [600, 601]. In this way, the toxicity of nanoparticles in vitro and in vivo, hemocompatibility, effect on major organs including liver and kidney, and metabolic pathways should be investigated [602]. The surface functionalization of nanoparticles is another important factor in cancer immunotherapy. The surface functionalization can affect the intracellular uptake of nanostructures and even their processing [603]. Furthermore, since nanoparticles have been applied for antigen capture, the surface charge, hydrophobicity and hydrophobicity can change the function of nanostructures in cancer immunotherapy [299]. Another factor is that the nanostructures can specifically target the lymphoid tissues or immune cells to enhance the potential of drugs in cancer immunotherapy [604]. Polymeric nanoparticles have shown high potential in vascular escape, infiltration into tissues and lymphatics and targeting lymphocytes, while liposomes show high uptake by phagocytic cells, including macrophages [603]. In addition, these nanoparticles demonstrate high safety along with capacity in cancer immunotherapy [605–608]. Notably, the nanoparticles can be considered as immune potentiates in which metal nanoparticles, virus-like nanostructures and other categories can stimulate immune responses through induction of B and T cells [603, 609] that is highly dependent on the size and surface charge of nanostructures [366, 610, 611]. However, carbon nanostructures have shown immunosuppressive impacts in some cases [612, 613]. Therefore, the introduction of nanoparticles for thr purpose of cancer immunotherapy is of importance.

## Data Availability

No datasets were generated or analysed during the current study.

## Abbreviations

ICIs: Immune Checkpoint Inhibitors
TME: Tumor Microenvironment
NFAT: Nuclear Factor of Activated T Cells
TOX: Thymocyte Selection-Associated High Mobility Group Box Protein
NR4A: Nuclear Receptor Subfamily 4 Group A
FDA: U.S. Food and Drug Administration
CTLA4: Cytotoxic T-Lymphocyte Antigen 4
PD-1: Programmed Cell Death Protein 1
PD-L1: Programmed Death-Ligand 1
TAMs: Tumor-Associated Macrophages
Th1: Type 1 T Helper Cell
IL-4, IL-10, IL-13: Interleukin-4, Interleukin-10, Interleukin-13
M1: M1 Macrophages
M2: M2 Macrophages
TGF-β: Transforming Growth Factor-Beta
α-SMA: Alpha-Smooth Muscle Actin
NK cells: Natural Killer Cells
IFN-γ: Interferon-Gamma
B cells: B Lymphocytes
T cells: T Lymphocytes
PD-1: Programmed Cell Death Protein 1
TCM: Central Memory T Cells
TEM: Effector Memory T Cells
TRM: Resident Memory T Cells
TSCM: Stem Cell Memory T Cells
TE: Effector T Cells
Th1: Type 1 T Helper Cell
IFN-γ: Interferon-Gamma
TCM: Central Memory T Cells
CEMIP: Cell Migration-Inducing Hyaluronidase 1
USP22: Ubiquitin-Specific Peptidase 22
RNF31: Ring Finger Protein 31
YAP: Yes-Associated Protein
CDKN2A/B: Cyclin-Dependent Kinase Inhibitor 2 A/B
SUSD6: Sushi Domain Containing 6
TMEM127: Transmembrane Protein 127
WWP2: WW Domain Containing E3 Ubiquitin Protein Ligase 2
mTORC1: Mechanistic Target of Rapamycin Complex 1
CD248: Cluster of Differentiation 248
OPN: Osteopontin
SERPINE1: Serpin Family E Member 1
RGS5: Regulator of G Protein Signaling 5
apCAFs: Antigen-Presenting Cancer-Associated Fibroblasts
rCAFs: Restraining Cancer-Associated Fibroblasts
iCAFs: Inflammatory Cancer-Associated Fibroblasts
PRDM1: PR Domain Zinc Finger Protein 1
ZnCDA: Zinc Chlorodipyridine Acetate
STING: Stimulator of Interferon Genes
ROS: Reactive Oxygen Species
TLR4: Toll-Like Receptor 4
MyD88: Myeloid Differentiation Primary Response 88
Fe3O4-SAS@PLT: Ferrimagnetic Mesoporous Silica Nanoparticles Loaded with Sulfasalazine and Functionalized with Platelets
CSF1: Colony Stimulating Factor 1
CSF1-R: Colony Stimulating Factor 1 Receptor
SIRPα: Signal Regulatory Protein Alpha
MCSF: Macrophage Colony-Stimulating Factor
MAPK: Mitogen-Activated Protein Kinase
PEGylated liposomes: Polyethylene Glycol-coated Liposomes
R848: Imiquimod analog
CCL5-siRNA: Chemokine (C-C motif) Ligand 5 Small Interfering RNA
Ce6: Chlorin e6
PFC: Perfluorocarbon
MIP-3β: Macrophage Inflammatory Protein-3β
Au@PG: Gold Nanoparticles coated with Polyaniline-Glucose
CaCO3: Calcium Carbonate
DGL-ZA: Dendrigraft Poly-L-Lysines-Zoledronate
MMP2: Matrix Metalloproteinase 2
cGAS/STING: Cyclic GMP-AMP Synthase/Stimulator of Interferon Genes
PLGA: Poly(lactic-co-glycolic acid)
CTL: Cytotoxic T Lymphocyte
MDSCs: Myeloid-Derived Suppressor Cells
Treg cells: Regulatory T cells
PARP: Poly(ADP-ribose) Polymerase
AMPKα/mTOR: AMP-Activated Protein Kinase Alpha/Mammalian Target of Rapamycin
GITR: Glucocorticoid-Induced Tumor Necrosis Factor Receptor
PLG: Poly(lactide-co-glycolide)
PLH: Poly-L-Histidine
IDO: Indoleamine 2,3-dioxygenase
STAT3: Signal Transducer and Activator of Transcription 3
STAT5: Signal Transducer and Activator of Transcription 5
mPEG2k-DSPE: Methoxy Polyethylene Glycol 2000-Distearoylphosphatidylethanolamine
iRGD: Internalizing RGD peptide
IR-780: Near-Infrared Fluorescent Dye
FGL1: Fibrinogen-Like Protein 1
mPEG2k: Methoxy Polyethylene Glycol 2000
ERCC1: Excision Repair Cross-Complementation Group 1
c-Myc: MYC Proto-Oncogene, BHLH Transcription Factor
OX40: Tumor Necrosis Factor Receptor Superfamily, Member 4 (TNFRSF4)
CpG: Cytosine-phosphate-Guanine
PL1: Phospholipid Nanoparticles 1
GITR: Glucocorticoid-Induced Tumor Necrosis Factor Receptor
PLG: Poly(lactide-co-glycolide)
PLH: Poly-L-Histidine
IDO: Indoleamine 2,3-dioxygenase
mPEG2k-DSPE: Methoxy Polyethylene Glycol 2000-Distearoylphosphatidylethanolamine
mPEG2k: Methoxy Polyethylene Glycol 2000
ERCC1: Excision Repair Cross-Complementation Group 1
CpG: Cytosine-phosphate-Guanine
PL1: Phospholipid Nanoparticles 1
AIEdots: Aggregation-Induced Emission dots
HMGB1: High Mobility Group Box 1
MART-1: Melan-A
CCL22: C-C motif chemokine ligand 22
LncRNA: Long Non-Coding RNA
